# Genistin: A Novel Estrogen Analogue Targeting ERβ to Alleviate Thrombocytopenia

**DOI:** 10.7150/ijbs.90483

**Published:** 2024-03-25

**Authors:** Xiaoqin Tang, Rui Liao, Ling Zhou, Taian Yi, Mei Ran, Jiesi Luo, Feihong Huang, Anguo Wu, Qibing Mei, Long Wang, Xinwu Huang, Jianming Wu

**Affiliations:** 1 Sichuan Key Medical Laboratory of New Drug Discovery and Druggability, Luzhou Key Laboratory of Activity Screening and Druggability Evaluation for Chinese Materia Medica, School of Pharmacy, Southwest Medical University, Luzhou,646000, China.; 2 School of Basic Medical Sciences, Southwest Medical University, Luzhou 646000, China.; 3 School of Pharmacy, Chengdu University of Traditional Chinese Medicine, Chengdu 611137, China.; 4 Education Ministry Key Laboratory of Medical Electrophysiology, Southwest Medical University, Luzhou 646000, China.

**Keywords:** Estrogen Analogue, Genistin, ERβ, thrombocytopenia

## Abstract

Thrombocytopenia, a prevalent hematologic challenge, correlates directly with the mortality of numerous ailments. Current therapeutic avenues for thrombocytopenia are not without limitations. Here, we identify genistin, an estrogen analogue, as a promising candidate for thrombocytopenia intervention, discovered through AI-driven compound library screening. While estrogen's involvement in diverse biological processes is recognized, its role in thrombopoiesis remains underexplored. Our findings elucidate genistin's ability to enhance megakaryocyte differentiation, thereby augmenting platelet formation and production. *In vivo* assessments further underscore genistin's remedial potential against radiation-induced thrombocytopenia. Mechanistically, genistin's efficacy is attributed to its direct interaction with estrogen receptor β (ERβ), with subsequent activation of both ERK1/2 and the Akt signaling pathways membrane ERβ. Collectively, our study positions genistin as a prospective therapeutic strategy for thrombocytopenia, shedding light on novel interplays between platelet production and ERβ.

## Introduction

Megakaryopoiesis, the intricate orchestration of lineage commitment, is modulated by a suite of molecules, with thrombopoietin (TPO) taking precedence as the MPL receptor ligand 1. Deficiencies in MPL expression manifest as congenital amegakaryocytic thrombocytopenia (CAMT) 2. The pivotal role of megakaryocytes in platelet production underscores the importance of both megakaryopoiesis and thrombopoiesis in guiding hematopoietic stem cells (HSCs) toward platelet formation. MPL deficiencies, either congenital in humans or through genetic manipulation in mice culminate in pronounced thrombocytopenia 2, 3. Current MPL agonists, while therapeutic agents, present challenges, including prohibitive costs and potential side effects 4, 5. Platelet transfusions, though vital for hemorrhage-prone patients, are constrained by donor availability, shelf-life, and contamination risks 6. Splenectomy, a surgical recourse, carries inherent risks and unpredictable outcomes, underscoring the pressing need for novel, efficacious thrombocytopenia treatments 7. Whereas medication is currently the mainstay of treatment for thrombocytopenia, side effects and price limit its use. Therefore, there is an urgent need for the discovery of a novel receptor agonist with high efficacy and low toxicity for the treatment of thrombocytopenia.

Recent literature has illuminated estrogen's nuanced role in hematopoiesis, with estrogen levels correlating with platelet counts 8. For example, clinical observations indicate that during puberty, as estrogen production in females escalates to support sexual maturation, females exhibit elevated platelet levels compared to males 9. Concurrently, estrogen facilitates the differentiation of megakaryocytes and influences the expression of estrogen receptors alpha and beta 10. It has been established that estrogen augments the polyploidization of megakaryocytes t via ERβ, and in murine thrombocytopenia models, it has demonstrated a platelet-increasing effect 11. Meantime, studies have reported that estrogen receptors (ERs or ESRs) have distinct effects on the fate of primitive and mature hematopoietic cells, as well as immune cells, leading to sex-based differences in hematopoietic activity 12-14. It is not clear whether this is due to differences in estrogen levels in the body. Therefore, it is necessary to understand the association of estrogen receptors with thrombocytopoiesis-related mechanisms. It is universally acknowledged that MPL have a number of downstream signaling pathways, including JAK2 ((Janus kinase) /STATs (signal transducer and activator of transcription), MAPK (mitogen-activated protein kinase) /ERK (extracellular signal-regulated kinases), and phosphatidylinositol 3-kinase (also known as phosphoinositide 3-kinase, PI3K) /Akt (protein kinase B) signaling pathway 15-18. The commonality of many current drugs for thrombocytopenia is to achieve a good therapeutic effect by activating these pathways. ER is mainly called nuclear receptor, but it is expressed in both the plasma membrane and cytoplasm. Estrogen-mediated signaling events can be divided into genomic and non-genomic events. Genomic effects involve the migration of estrogen-receptor complexes to the nucleus and interact directly with chromatin to elicit effect 19. Non-genomic effects, on the other hand, involve indirect regulation of gene expression through various intracellular signaling events, including downstream pathways of MPL. Yet, the precise mechanism underlying platelet production and its association with estrogen receptors remains elusive.

At present, the fusion of artificial intelligence (AI) with cutting-edge experimental methodologies promises accelerated drug discovery 20. Leveraging this, our study employed a randomForest-based model, identifying genistin (Gen) as a potential therapeutic compound. Found in diverse dietary sources, genistin's myriad health benefits are well-documented 21-23. Intriguingly, as a phytoestrogen, genistin shares structural parallels with both natural and synthetic estrogens 24. Significantly, our findings indicate that genistin enhances megakaryocyte differentiation and has an effect on estrogen receptors. However, its pharmacodynamic effect and specific mechanism need to be further elucidated. Therefore, we aimed to determine whether administration of genistin can promote platelet production, thereby improving platelet count recovery after radiation using mouse models, and to investigate mechanisms *in vitro*. This study was systematically revealed that genistin facilitated the production and maturation of megakaryocytes, as well as PPF and platelets. And thrombosis promoted by genistin contributes to the recovery of platelets in radioactive mice with thrombocytopenia. On the mechanism section, we found that this protective effect on thrombopoiesis was associated with the role of genistin in regulating both ERK1/2 and Akt signaling pathway. Meanwhile, both pathways are downstream pathways where genistin activates membrane ERs. Therefore, our findings further support the role of ERβ in hematopoiesis and their potential therapeutic effects and suggest that genistin may be a clinical candidate for the treatment of radioactive thrombocytopenia.

## Materials and Methods

### Construction of ML-based drug screening model

The virtual screening model was established as described in the previous study 25. Specifically, as a virtual screening flowchart shown in Figure A. First, we set up a database that collected 34 small molecule compounds that could promote MK differentiation or platelet production and 740 small molecule compounds that had no obvious effect on MK differentiation and platelet production. We then download SDF files with the corresponding 2D structural formula for each compound in the PubChem Compound database of the National Center for Biotechnology Information (https://www.ncbi.nlm.nih.gov) via its CAS Number 26. Then, using the software Mold2 (https://www.fda.gov/science-research/mold2), we obtained 777 molecular descriptors for each compound, with active compounds labeled 1 and inactive compounds labeled 0. Molecular Descriptors contain several molecular properties, such as the Electrotopological index (D197), Molecular weight (D197), and aromatic bonds ratio (D776). Molecular descriptors for 2 active and 10 inactive small molecule compounds were then randomly selected from the database as the validation set, and the remaining 762 small molecule compounds were used as the training set. Since the proportion of samples of active and inactive compounds in the training set is severely imbalanced, a synthetic minority oversampling technique (SMOTE) is used to balance positive and negative samples 27. The active compounds in the training set changed from 32 to 672, the inactive compounds from 730 to 699, and the ratio of active compounds to inactive compounds from 1:22.81 to 1:1.04. Using the software RStudio (https://www.r-project.org/), the Gini index of each feature is calculated using randomForest packages and the importance of the features is ranked according to the Gini index 28. In order to avoid affecting the results of the virtual filter, it is necessary to delete all the features with a Gini index of zero, leaving 554 features. Molecular descriptors with 50%, 60%, 70%, 80%, 90% and 100% feature importance, were used to generate 6 training sets, and then were input 6 training sets into the randomForest model of Orange software (https://orangedatamining.com/). According to the area under the ROC. The value of curve (AUC) was selected the optimal training set to train randomForest, the validation set was used to validate the model, and finally the compound was predicted 29. Finally, the model was used to predict the drug activity of 1012 natural products from our laboratory's own natural product library, including carbohydrates and glycosides, phenylpropanoids, quinones, flavonoids, terpenoids, steroids, alkaloids, phenols, acids, and aldehydes.

### Chemical

Genistin (CAS: 529-59-9, purity≥99.78%, as determined by HPLC) was obtained from Chengdu Push Biotechnology Co., Ltd (Chengdu, China).

### Cell culture

Meg-01 (human megakaryocytic leukemia cell line) and K562 (chronic myelogenous leukemia cells) cells were purchased from American Type Culture Collection (Bethesda, MD, USA) and cultured with RPMI-1640 medium (Gibco, Invitrogen Corporation, Carlsbad, CA, USA) supplemented with 10% fetal bovine serum (Sperikon Life Science & Biotechnology Co., Ltd., Chengdu, China) and 1% penicillin/streptomycin (Gibco, Invitrogen Corporation, Carlsbad, CA, USA). The cells were incubated in a humidified atmosphere with 5% CO_2_ at 37 °C.

### CCK-8 assay

Cell proliferation of Meg‑01 and K562 cells was evaluated by Cell Counting Kit‑8 (CCK‑8) assay, according to the manufacturer's instructions (Dojindo, Kyushu, Japan). In brief, 4 × 10^3^ cells/well were seeded in 96-well plates and then were treated for 1, 3 and 5 days with or without genistin (5, 10 and 20 μM) at 37 °C and 5% CO2. The untreated cells were considered the control group. And then 10 μL of CCK‑8 solution was added to each well and incubated at 37 °C for 2-4 h. The absorbance (OD) was measured at 450 nm using a microplate reader (BioTek, IL, USA).

### Lactate dehydrogenase (LDH) assay

The LDH assay of Meg‑01 and K562 cells was performed using an LDH assay kit (LEAGENE, Beijing, China) in accordance with the manufacturer's protocol. Briefly, the cells were planted in 96-well plates (5 × 10^3^ cells/well) and treated for 1, 3 and 5 days with or without genistin (5, 10 and 20 μM) at 37 °C and 5% CO_2_. And then the cytotoxicity was measured by an LDH cytotoxicity assay kit (Beyotime, Jiangsu, China) in accordance with the manufacturer's instructions.

### Giemsa staining

After treatment with or without genistin (5, 10 and 20 μM) for 5 days, the cells were harvested and washed with PBS for twice. Then the cells were fixed with fixing solution (methanol:glacial acetic acid=3:1 (v/v)), and stained with Giemsa solution (Solarbio, Beijing, China) for 8 min. Finally, the stained cells were photographed under electron microscope (20×).

### Phalloidin staining

After treatment with or without genistin (5, 10 and 20 μM) for 5 days, the cells were immobilized at room temperature with 4% paraformaldehyde solution (Biosharp, Anhui, China, 1810473) in phosphate-buffered saline (PBS). The cells were rinsed with PBS and permeated with 0.5% Triton X-100 solution. After washing again with PBS, TRITC-labeled phalloidin working solution was allowed to infiltrate into the cells on glass slides. The cells were incubated in the dark at room temperature and then washed again with PBS. The nuclei were restained with DAPI solution (100 nM). The images were observed under a fluorescence microscope (Nikon Ts2R/FL, Japan).

### Analysis of MK differentiation

A total of 4 × 10^4^ cells were seeded into a 6-well plate and treated with genistin (5, 10, and 20 μM), PHTPP (20 μM) (MedChemExpress, Monmouth Junction, NJ), LY294002 (10 μM) (MedChemExpress, Monmouth Junction, NJ), or SCH772984 (10 μM) (MedChemExpress, Monmouth Junction, NJ) for 5 days. The cells were harvested and washed once by ice-cold PBS. 5 μL of FITC conjugated anti-CD41 and PE conjugated anti-CD42b (Biolegend, San Diego, CA) were added to the cells and incubated on ice for 20 min in the dark. The labeled cells were washed twice and resuspended in 500 μL of PBS, subsequently analyzed by the BD FACSCanto II flow cytometer (BD Biosciences, San Jose, CA).

### Polyploidy analysis

A total of 4 × 10^4^ cells were seeded into a 6-well plate and treated with genistin (5, 10, and 20 μM) and PHTPP (20 μM) (MedChemExpress, Monmouth Junction, NJ) for 5 days. The cells were collected, washed with PBS twice and permeabilized with ice-cold 70% ethanol at 4 °C until use. According to the manufacturer's protocol, the cells were washed twice and then treated with 100 μL trypsin inhibitor/RNase buffer using the CycleTEST™ PLUS DNA Reagent Kit (BD Biosciences, San Jose, CA, USA). After that, the cells were incubated with 300 μL PI stain solution for 10 min. Finally, the ploidies of the samples were detected by a BD FACSCanto II flow cytometer (BD Biosciences, San Jose, CA, USA) and analyzed by FlowJo software.

### Cell apoptosis assay

An Annexin V-FITC/PI Apoptosis Detection kit (BD Biosciences, San Jose, CA, USA) was used to determine the apoptotic effect of genistin according to the manufacturer's instructions. Cells were incubated in 6-well plates at a density of 4 × 10^4^ cells/mL with or without genistin (5, 10 and 20 μM) treatment for 5 days. After treatment, the cells were harvested, washed twice with cold phosphate buffer saline (PBS) and resuspended in 100 µL binding buffer. Next, 5 μL Annexin V-FITC and propidium iodide (PI) were added to the cell suspension and incubated at room temperature for 15 min in the dark. Cells were immediately analyzed by BD FACSCanto II flow cytometer (BD Biosciences, San Jose, CA, USA) and analysis was performed by FlowJo software.

### Animals and drug administration

Specific pathogen-free (SPF) Kunming (KM) mice, 2 months old and 18-22 g in weight, were purchased from Da-suo Biotechnology Co. Ltd., (Chengdu, Sichuan, China). The mice were maintained under a specific pathogen-free condition and given free access to standard food and purified water. All the operations on mice were in compliance with guidelines approved by the laboratory animal ethics committee of the Southwest Medical University (Luzhou, China). After acclimation for a week, all mice were randomly divided into six groups: normal control group, thrombocytopenia model group, TPO positive group (3000 U/kg/d), genistin group (2.5, 5.0, 10.0 mg/kg/d), and normal genistin group (10 mg/kg/d). In addition to the normal control group and normal genistin group (10 mg/kg/d), the mice of other groups were given a single dose of 4 Gy X-ray total body irradiation (TBI). After irradiation, both of the normal control group and model group were intraperitoneally administered with normal saline for 12 consecutive days. The other groups were intraperitoneally administered with TPO or different doses of genistin (2.5, 5.0, 10.0 mg/kg/d) for 12 consecutive days, respectively.

### Hematological analysis

A small amount of peripheral blood (PB) (40 μL) was drawn from fundus vein plexus on the indicated days (on days 0, 4, 7, 10, 12) and treated with 160 μL of diluent according to the instrumental assay. The hematologic parameters were then measured by the automatic blood cell analyzer (Sysmex XT-1800i/2000IV; Kobe, Japan).

### Flow cytometry analysis of BM, spleen, lung and blood cells

All BM cells were flushed out from the femur by normal saline and the spleen and lung was ground into single cells and filtered by nylon net (106 μm). For analysis of MKs in peripheral blood, 50 µL of blood was taken from the ophthalmic venous plexus and added to an EP tube prefilled with sodium citrate for mixing. After being washed with PBS twice, the cell density was adjusted to 1 *×* 10^6^ per sample by counting on a hematology analyzer. Then, according to the purpose of the experiment, the samples were labeled with the corresponding antibodies for 30 min in the dark, respectively. It is worth noting that if PI is required for relabeling, 300 µL PI stain solution is incubated for another 15 min after completing other antibody labeling. Finally, marker expression was analyzed using BD FACSCanto II flow cytometry (BD Biosciences, San Jose, CA, USA). Specific flow antibody information were as follows: FITC conjugated anti-CD41 (BioLegend, San Diego, CA, USA), PE‑conjugated anti‑CD117 (c‑Kit, BioLegend, San Diego, CA, USA), PE‑conjugated anti‑CD61 (BD Biosciences, San Jose, CA, USA), APC conjugated anti-CD62p (BioLegend, San Diego, CA, USA), FITC conjugated anti-CD42d (BioLegend,y,San Diego, CA, USA).

### Hematoxylin & eosin staining and Immunohistochemical staining

After the mice were treated with genistin for 10 days, the femurs, spleens, liver, kidney and thymus were separated from randomly selected 4 mice of each group. The femurs were fixed in 10% formaldehyde over 24 h, followed by decalcification with decalcifying solution for more than 1 month. Then the femurs and above organs were embedded by paraffin and cut into 5 µm thick sections. The samples were then stained with hematoxylin and eosin (H&E) or anti-VWF antibody (Proteintech, 1L, USA, 11778-1-AP, 1:100). Images were captured using an Olympus BX51 microscope (Olympus Optical, Japan). Three fields of each sample were randomly shot and the number of MKs was counted.

### Tail bleeding assay

Tail bleeding time was detected as described in the literature report 30. Specifically, tails of anesthetized mice were cut 0.5 cm from the tip and immediately immersed in saline (37 °C). The time taken for the bleeding to stop (no blood flow for 1 minute) was recorded. Tail-bleeding assays were stopped at 600 seconds if the bleeding did not stop.

### Measurement of thrombosis *in vivo*

Arterial arterioles were injured with 10% ferric chloride (FeCl_3_), as previously described 31, 32. Briefly, the left carotid arterie of anesthetized mice was exposed and the thrombosis was induced by topically applying a piece of filter paper (1 x 2 mm) saturated with 7.5% FeCl_3_ solution directly on the carotid artery for 1 min. After removing the filter paper and washing with PBS, the blood flow was continuously monitored with a vascular flow probe using a Transonic Model TS420 flowmeter (Transonic Systems, Ithaca, NY, USA).

### Platelet isolation and sample preparation

Platelet extraction and isolation were performed as described previously 32. An anticoagulant tube containing 3.8% sodium citrate (V:V = 1:9) was filled with mouse blood. Blood was immediately centrifuged at 100× g for 10 min at room temperature to thoroughly separate the platelet-rich plasma (PRP). To segregate platelets, PRP was centrifuged at 400× g for 10 min at 22 °C. The platelet pellet was cleaned and resuspended in modified Tyrode's buffer (137 mM NaCl, 2.9 mM KCl, 0.34 mM Na_2_HPO_4_, 12 mM NaHCO_3_, 5 mM HEPES, 5 mM glucose, 1 mM MgCl_2_, and 1 mM CaCl_2_, pH 7.3) for the subsequent detection while the supernatant was pipetted away and discarded. They were then given permission to relax for 1-2 hours at 25 °C.

### Platelet activation analysis

Platelet activation was performed using washed platelets prepared as described above and stimulated with agonist (ADP: 10 µM, Helena Laboratories, USA) for 10 min at 37 ◦C, after which resting or activated platelets were incubated with PE‑conjugated anti‑CD62p (BioLegend, San Diego, CA, USA) for 20 min at room temperature in the dark by flow cytometry analysis.

### Platelet aggregation

Platelet aggregation was performed as described previously 32. The prepared platelets isolated in method 2.17 above were taken, the platelet count was calculated using a hematology analyzer, and the platelet density was adjusted to 2 × 10^8^ platelets/mL. Finally, the platelets were stimulated by ADP (helena Laboratories, USA) and platelet aggregation was analyzed using a turbidimetric aggregation-monitoring device (Helena Laboratories, Beaumont, TX, United States).

### Platelet adhesion

For the preparation of plates coated with collagen, 250 μL of 5 μg/mL collagen (Helena Laboratoriese Laboratories, USA) was added to the bottom of each confocal dish and kept overnight at 4 ℃. After washing with PBS, each well was blocked with 1% BSA for 1 h at room temperature. PRP (2× 10^7^ cell/mL) was then added to each well and incubated at 37 °C for 45 min. After unbound platelets were removed by PBS, wells were fixed with 4% paraformaldehyde for 15 min and permeabilized with 1% Triton X-100 for 10 min at room temperature. After that, the adhering platelets were stained with TRITC-conjugated phalloidin (1:200) 33. The representative images were captured using the inverted fluorescence microscope (Nikon Ts2R/FL, Japan). The average coverage of adhering platelets was calculated by ImageJ software.

### Clot retraction

For clot retraction, 400 μL of washed platelets or whole blood was mixed with 1200 μL of normal saline, and calcium chloride (2 mM) and thrombin (1 U/mL) were added. In addition, platelet contraction required additional intervention with fibrinogen (1 mg/mL). Finally, the mixture was incubated at 37 °C for different time intervals and pictures were taken. The degree of platelet clot retraction was analyzed using ImagJ software and mass measurements were performed on plaques retracted from whole blood.

### Molecular docking simulation and molecular dynamics simulation

Molecular docking is one of the common methods used in structure‑based drug design. When small molecule ligands bind to target molecules to form stable complexes, the preferred concept can be predicted, and the binding strength and affinity between them can be calculated. It is widely used to better understand the interaction characteristics of small molecules with therapeutic targets of many pathogens of clinical interest. Here, molecular docking was used to predict the binding affinity between genistin and potential molecular targets. The 3D structure of genistin was obtained from PubChem. The 3D crystal structures of human potential molecular target (ERβ) were obtained from the RCSB Protein Data Bank (https://bivi.co/visualization//rcsb‑protein‑data‑bank) on 12 January 2023. The optimized structure of the targets was then obtained by adding hydrogen atoms, adding charge and mini‑ mizing energy. The molecular docking between genistin and ERβ was simulated using SYBYL‑x 2.0 and visualized using PyMOL software. The Surflex‑Dock score (total score) indicates the binding affinity. Finally, the total score is arranged from high to low. The higher the binding score, the more likely it is to be its drug target, and then the following related experiments are used for verification.

### Drug Affinity Responsive Target Stability Assay (DARTS)

Meg-01 cells were collected, lysed with RIPA lysis buffer on ice for 15 min, and centrifuged at 12,000 rpm/min for 15 min, and the supernatant was obtained. The protein concentration was determined by the Bradford reagent. Before drug treatment, the protein was divided into 2 portions. The samples were treated with genistin and DMSO (Solarbio, D8371, Beijing, China) at room temperature for 6 h. Then, the two parts were divided into 3 parts. Thereafter, different volumes of proteinase E (0.05 mg/mL) were added according to the mass ratio of proteinase E to protein mass (1:500, 1:1000, and 1:1500) and incubated at 40 °C for 10 min. Finnaly, 5× loading buffer was added and boiled for 10 min. All parts of each sample were used for western blot analysis. Another detection method is roughly the same as above, except that each sample controls the same enzyme concentration, incubates at different genistin concentrations, and detects its sample expression.

### Cellular thermal shift assay (CETSA)

Meg-01 cells were incubated with or without genistin (200 μM) for 4 h, and then the cells were collected and subjected to CETSA assay 34. Briefly, incubated cells were equally divided into 10 parts, each part was heated for 3 min under different temperatures (45, 48, 51, 54 and 57 °C), and then the heated cells were kept at -80 °C for 12 h, and then at room temperature for 5 min. After that, cell lysates were extracted by centrifugation at 12,000× g for 15 min. Levels of ERβ were assessed by western blot.

### Immunofluorescence assay

Meg-01 cells were collected after drug intervention and fixed with 4% paraformaldehyde fixative solution at room temperature. Subsequently, we washed the cells three times with PBS, added 0.5% Triton X-100 to perforate the cell membranes within 10 min, and blocked this process by adding 5% BSA at room temperature. Then, anti-GATA-1 and anti-NF-E2 antibodies were incubated with the cells overnight at 4 °C. After washing with PBS, the cells were coincubated with DAPI to observe the nuclear translocation of the transcription factors GATA-1 and NF-E2 via fluorescence confocal microscopy.

### Western blot

Cells that had undergone various treatments with or without genistin (5, 10 and 20 μM) for 5 days were lysed with 1× RIPA lysis buffer (CST, MA, USA) containing protease inhibitor cocktail. Protein concentration was determined by the Quick StartTM Bradford 1× Dye Protein Assay Reagent (Bio-Rad, CA, USA). Equal amount of protein (25 μg per sample) was loaded and separated by SDS-PAGE. After electrophoresis, the proteins on the SDS-PAGE were transferred onto a polyvinylidene fluoride (PVDF) membrane which was then blocked with 10% no-fat milk in TBST for 1 h at room temperature. The membrane was then incubated with the primary antibodies overnight at 4 °C. After incubation, the membrane was further incubated with horseradish peroxidase (HRP)-conjugated secondary antibodies for 1 h at 37 °C. Finally, the bands on the membrane were revealed by UltraSignal Hypersensitive ECL Chemiluminescent Substrate (4A Biotech Co., Ltd., China) and detected by the ChemiDoc MP Imaging System (Bio-Rad, California, USA). Band intensities were quantified by using ImageJ software (NIH, USA), and the relative expression of the proteins of interest to GAPDH were calculated. The primary antibodies were as follows: ERβ (Affinity, AF6469), HSP90 (Abmart, T55568S), p-ERK (CST, 4370S), ERK (CST, 4696S), p-MEK (CST, 9154T), MEK (CST, 4694S), p-PI3K (Abmart, TA3241), PI3K (Abmart, TA5112), p-AKT (Abmart, TA0016S), AKT (Abmart, TU421951S), GATA1 (CST, 3535S), NF-E2 (Proteintech, USA, 11089-1-AP), RUNX1 (Proteintech, USA, 25315-1-AP), EGR1 (Proteintech, USA, 22008-1-AP) and GAPDH (Proteintech, USA, 60004-1-lg).

### Statistical analysis

All data presented as means ± SD were analyzed by using GraphPad Prism 9.0 Software (San Diego, CA, USA). Student's t-test or TWO-way ANOVA analysis was applied for statistical analysis to compare all the different groups in the current study. The difference was considered to have statistical significance if P <0.05.

## Results

### Construction of drug screening model based on randomForest

**Figure 1:**
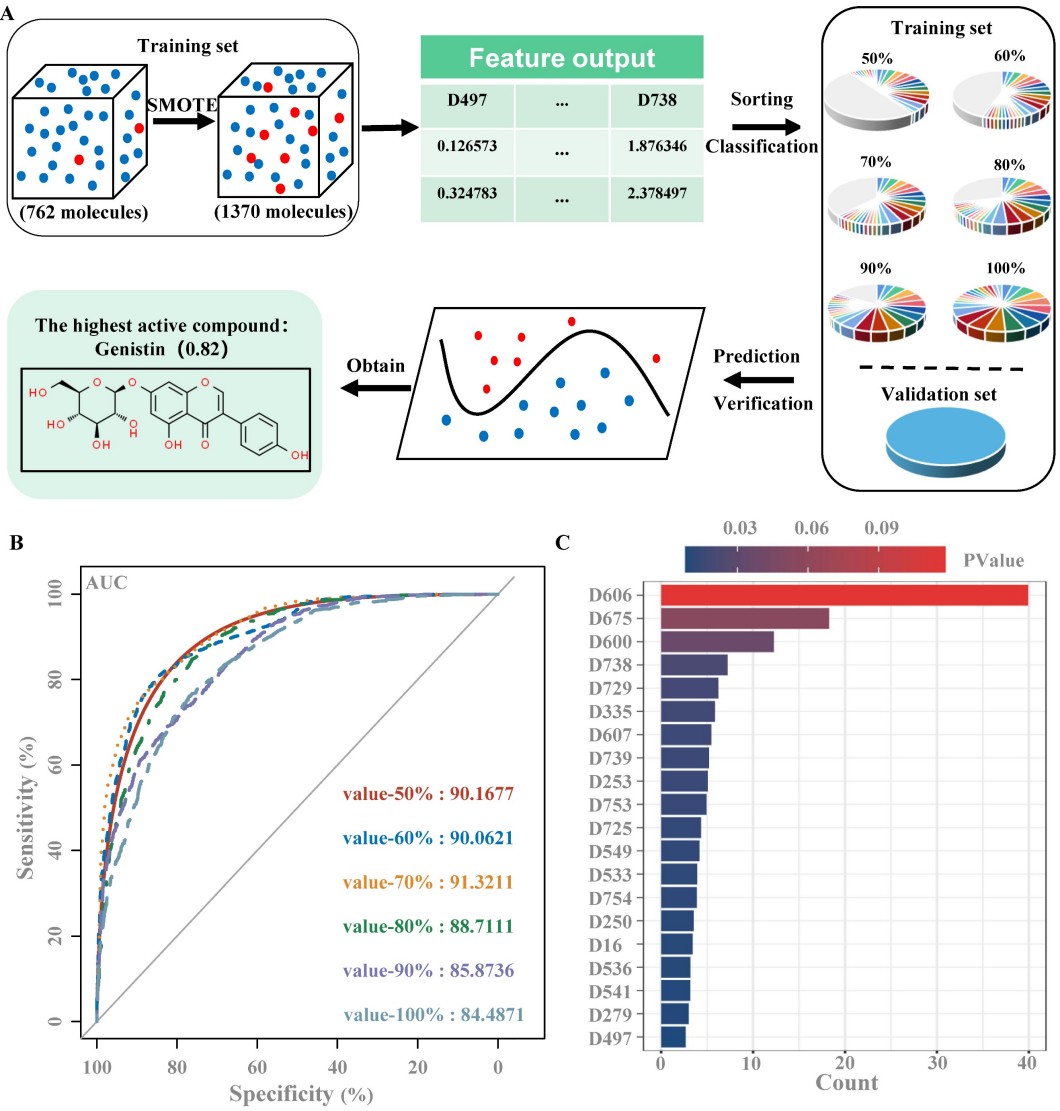
**Construction of drug screening model based on randomForest (A)** Flow chart of virtual screening; **(B)** ROC curves of six training sets; **(C)** The top 20 molecular descriptors of the training set with an importance ratio of 70%.

In this study, we used randomForest to build a virtual drug screening model. Six training sets were established, and the dataset with an importance ratio of 70% was found to have the best prediction performance, with an area under the curve (AUC) value of 0.913 (Figure 1B), and a total of 89 features (Figure 1C). Then, the validation set is used to detect the performance of the model, with a threshold of 0.5, and the results show that the prediction accuracy of the model is 91.7%. Finally, the model was used to conduct virtual screening of active drugs in the natural product library, and the final score of gentylenin was 0.82, which had potential activity.

### Genistin dose-dependently promotes MK differentiation and maturation *in vitro*

**Figure 2:**
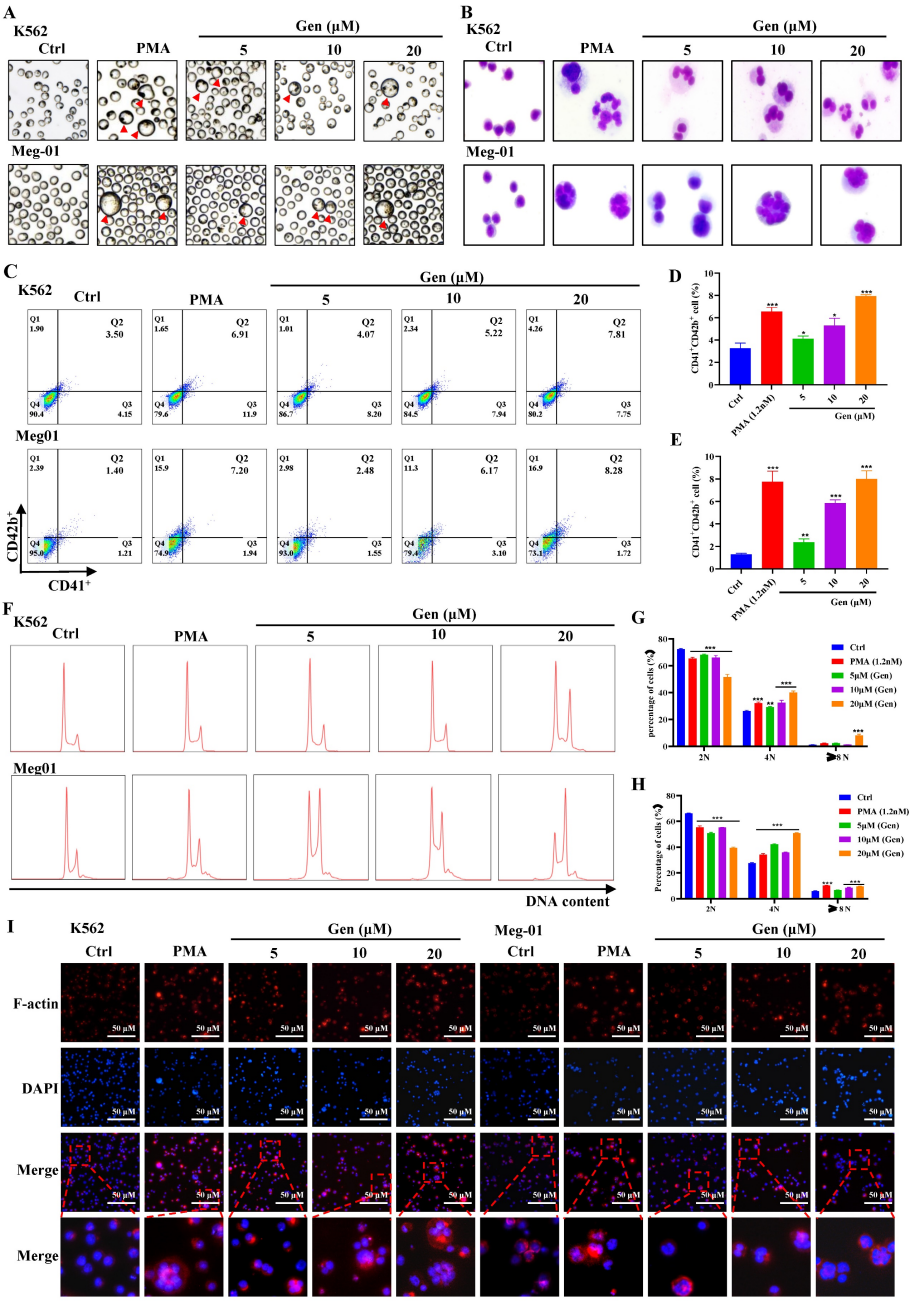
** Genistin dose-dependently promotes MK differentiation and maturation *in vitro*. (A)** Representative images of K562 and Meg‑01 cells treated with PMA or genistin treatment for 5 days, the red triangle represents large megakaryocytes; **(B)** K562 and Meg-01 cells treated with PMA or genistin treatment for 5 days and stained with Giemsa; **(C-E)** The expression of CD41 and CD42b was detected by flow cytometry after the K562 and Meg-01 cells were treated with PMA and genistin for 5 days. The histogram shows the percentage of CD41^+^CD42b^+^ cells in each group (n=3); (F-H) PI staining in K562 and Meg‑01 cells was used to analyze DNA content (2, 4 and ≥8 N) by flow cytometry after PMA and genistin intervention for 5 days (n=3). On the right, this statistical histogram displays cell proportions; (I) Immunofluorescence analysis of the expression of F-actin after PMA and genistin intervention for 5 days in K562 and Meg‑01 cells (n=3). F-actin was visualized by red fluorescence and cell nucleus was stained with DAPI (blue). All data represents the mean ± SD of three independent experiments; compared to the control group. ^*^p < 0.05, ^**^p < 0.01, ^***^p < 0.001, vs the Ctrl group. PMA: phorbol-12-myristate-13-acetate. Gen: genistin.

First, Meg-01 and K562 cells were given varying dosages of genistin to determine whether it had any direct effects on MK differentiation and maturation. Meanwhile, we selected PMA as a positive drug for MK differentiation *in vitro* 35, 36. After treatment for 5 days, we found that plenty of MK-like big cells increased with concentration in the genistin-treated groups, while seldom appeared in the control group (Figure 2A). Then, we evaluated the safety and activity of Meg‑01 and K562 cells treated with genistin for the specified period to establish the ideal concentration for MK *in vitro*. Combined with cell viability, cytotoxicity, and apoptosis experiments (Figure S1), we selected 5, 10, and 20 µM genistin for subsequent detection.

To further determine the presence of genistin on MK to promote differentiation, we conducted additional experiments *in vitro* studies. Megakaryocyte development is a process of "excessive hypertrophy", including the expansion of a large number of cell volumes, the formation of polyploidy, and the formation of the demarcation membrane system (DMS) 37. MKs with increased size and multilobulated nuclei were frequently observed after treatment with genistin for 5 days (Figure 2A-B). MK differentiation was characterized using the CD41 and the CD42b expression levels on the cell membrane and increased DNA content (polyploidization). CD41 is a specific surface antigen of MKs, Meanwhile, CD42b is an expression marker for megakaryocyte maturation 38. After treatment for 5 days, the expression of the MK marker CD41 and CD42b increased in a dose-dependent manner in these cells (Figure 2C-E). Similarly, a dose-dependent increase in the ploidy induced by genistin was observed in these cells (Figure 2F-H). Platelet genesis is driven by microtubule assembly and reorganization. MK maturation and proplatelet formation require dynamic reorganization of the actomyosin cytoskeleton and microtubules 39. Therefore, we performed clinocyclic peptide staining. Phalloidin staining revealed that these MK-like cells in the genistin-treated groups exhibited increased size and bviously multilobulated multinuclear, while the control group showed low nuclear level (Figure 2I). What's more, genistin treatment significantly increased the expression and induced an aggregation of F-actin, which might be conducive to proplatelet formation. Taken together, above data suggest that genistin has the ability to promote megakaryocyte differentiation and maturation.

### Genistin promotes the expression of megakaryocyte differentiation-related transcription factors

**Figure 3:**
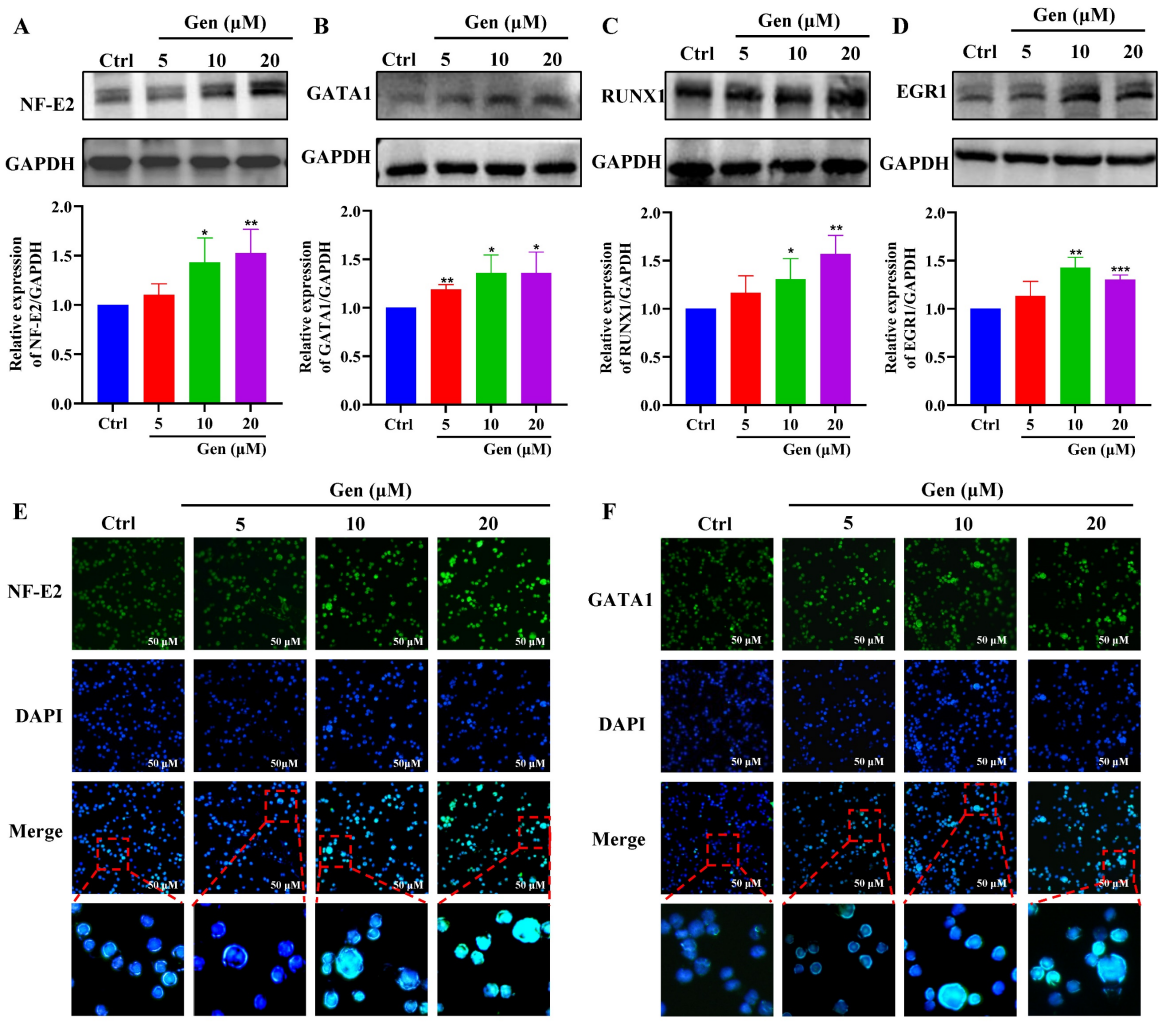
** Genistin promotes the expression of megakaryocyte differentiation-related transcription factors; (A-D)** Randomly selected transcription factors were detected by western blot; **(E-F)** Representative immunofluorescence image of the nuclear translocation of NF‑E2 and GATA1 in Meg‑01 cells upon treatment with genistin for 5 days; Data represent the mean ± SD of three independent experiments. ^*^p < 0.05, ^**^p < 0.01, ^***^p < 0.001, vs the Ctrl group. Gen: genistin, Ctrl: control.

To further clarify the role of genistin in megakaryocyte differentiation, we examined the expression of transcription factors. First, hematopoietic cell fate and megakaryocyte differentiation are largely coordinated by the temporal expression of various transcription factors 40. Therefore, our randomly selected transcription factors were detected by western blot, and the results showed that their expression was significantly upregulated after genistin intervention (Figure 3A-D). Among these transcription factors, the transcription factor NF-E2 is required during the terminal stage of megakaryocyte maturation and proplatelet production 41. And GATA1 is largely studied for its requirement in erythropoiesis and activation of globin gene expression but is also essential for megakaryopoiesis and transcription of the gene encoding megakaryocyte-specific receptor glycoprotein IIb 42-44. Then, to observe a visual change, we proceeded to these two transcription factors to proceed with immunofluorescence to detect changes in their expression. The results were consistent as expected (Figure 3E-F). The above studies further show that genistin promotes megakaryocytes differentiation and promotes the expression of transcription factors *in vitro*.

### Genistin administration increases circulating platelets

**Figure 4:**
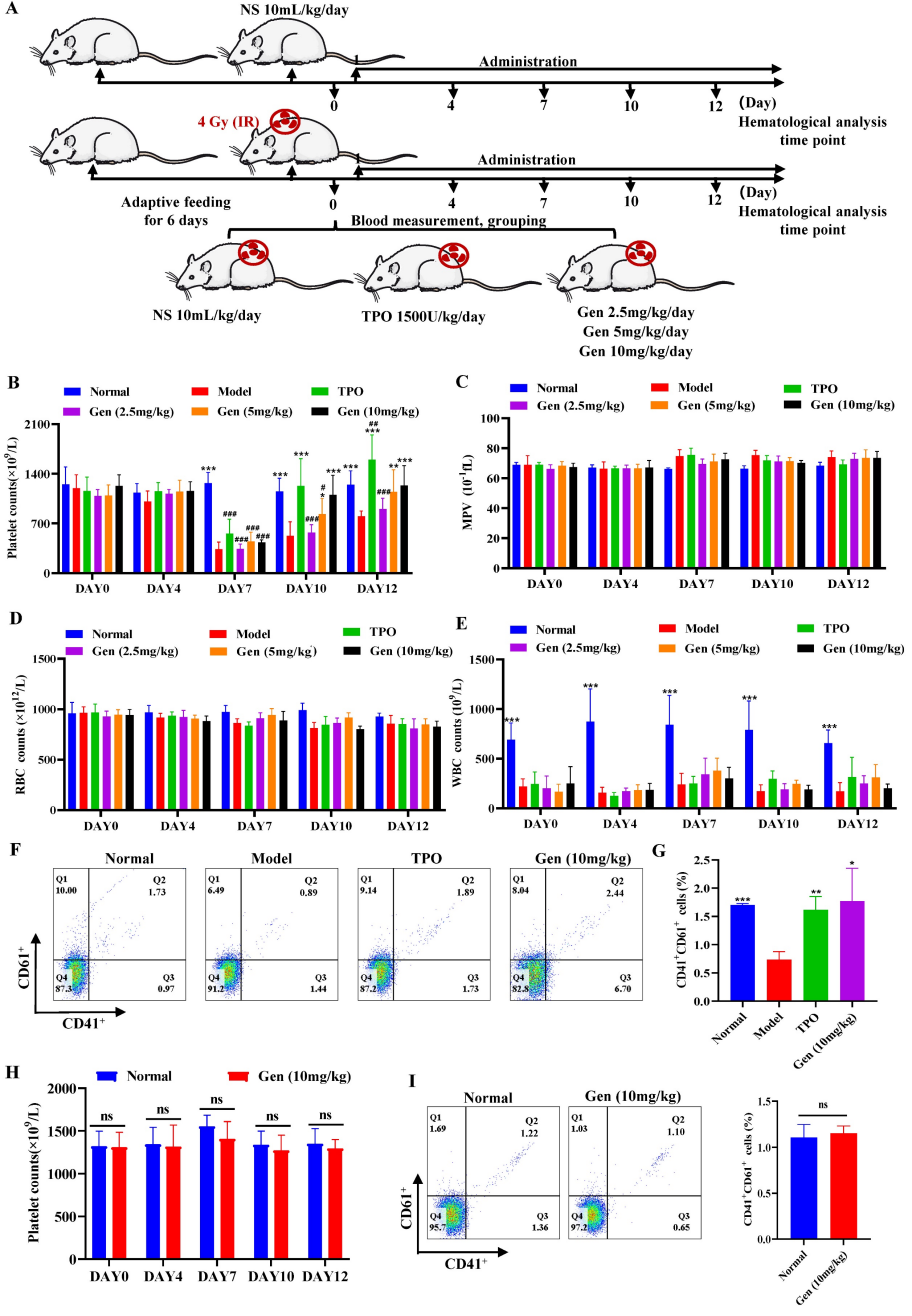
** Genistin administration increases circulating platelets (A)** Schematic diagram of animal experiment. **(B-E)** The number of platelets, WBC, RBC and MPV were counted in peripheral blood of each group on day 0, 3, 7, 10, and 12. Each group contains 12 mice (6 male mice and 6 female mice). **(F)** The expression of CD41 and CD61 was detected by flow cytometry in the peripheral blood of each group (n=3). **(G)** The histogram indicates the percentage of CD41^+^CD61^+^ cells in each group (n=3). **(H)** Platelet changes during normal administration in mice. Each group contains 12 mice (6 male mice and 6 female mice). **(I)** The expression of CD41 and CD61 was detected by flow cytometry in peripheral blood of control groups and normally administered (n=3). The histogram indicates the percentage of CD41^+^CD61^+^ cells in each group. The data represent the mean ± SD of three independent experiments; ^*^p < 0.05, ^**^p < 0.01, ^***^p < 0.001, vs the model group. ^#^*p* < 0.05, ^##^*p* < 0.01, ^###^*p* < 0.001, vs the normal group. Gen: genistin.

Since *in vitro* studies have found that genistin can promote the differentiation of megakaryocytes *in vitro*, we next constructed a radiation-damaged thrombocytopenia model (RIT) mice to investigate whether genistin has the ability to improve platelet count *in vivo*. We first injected RIT mice with genistin (10 mg/kg) to verify its role in thrombopoiesis promotion *in vivo*. According to the purpose of the experiment, the control variable method was used to group and administer the mice (Figure 4A). Among them, a mouse model of thrombocytopenia is given systemic radiation at a single dose of 4.0 Gy. As the results showed, after irradiation on day 0, the level of white blood cells decreased rapidly and significantly compared with that of control mice, indicating that the RIT mice were successfully constructed (Figure 4E). As expected, on day 0, the basal numbers of peripheral blood platelets in experimental mice were approximately 1050 × 10^9^/L, and decreased after irradiation. The platelet counts of the irradiated mice reached a nadir (430 × 10^9^/L) on day 7, but recovered gradually. There was no difference in platelet counts among the five groups on day 0 or day 7.

Recovery was better in the TPO and genistin-treated group than in the saline control group (Figure 4B). Additionally, the results showed that the WBC, RBC and MPV values of the experimental group did not change significantly compared with the model group, which indicated that genistin did not have an effect on other cells at these concentration (Figure 4C-E). To further confirm that the genistin-group accelerated platelet recovery compared with model mice, we selected the genistin dosing group with the best effect to perform CD41/CD61 flow cytometry analysis on peripheral blood cells. The CD41^+^CD61^+^ ratio in peripheral blood cells was significantly higher in the genistin‑treated and TPO groups (Figure 4F-G). Notably, we compared platelet counts in the genistin-treated model group (after recovery) to the normal group with continued drug administration. The result revealed that by day 10, both TPO and genistin groups reached normal platelet levels compared to the control (Figure 4B).

However, upon further administration, TPO continued to significantly elevate platelets, while genistin-treated mice maintained normal levels, mirroring the results in non-thrombocytopenic mice (Figure 4B). This supports our hypothesis that genistin primarily benefits thrombocytopenic states, minimizing the risk of platelet overproduction in healthy individuals. Therefore, to further detect whether genistin has an effect on normal platelet counts in mice, we administered genistin to normal mice. Hemaatological analysis and peripheral blood flow cytology showed no significant difference between the two groups (Figure 4H-I). This suggests that genistin has the potential to regulate platelet homeostasis, but its specific effects and mechanisms need to be further studied. The above data suggest that genistin, similar to TPO, had radioprotective effect on platelets. And it does not increase platelet counts in normal mice relative to TPO.

### Genistin promotes megakaryocyte production and differentiation *in vivo*

**Figure 5:**
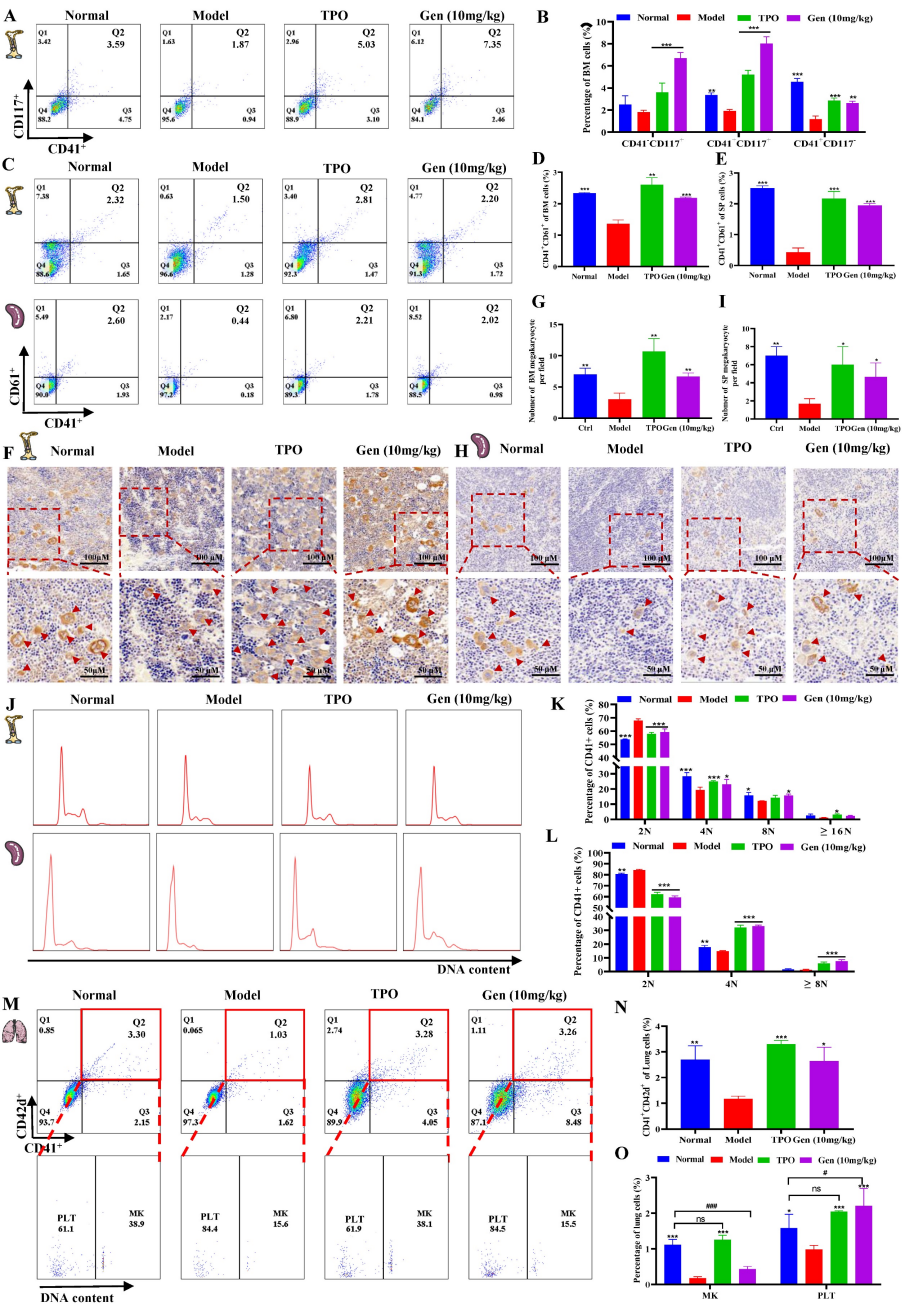
** Genistin promotes megakaryocyte production and differentiation *in vivo*. (A-B)** Flow cytometry analysis of CD117 and CD41 expression of each group after genistin treatment for 12 days. The histogram represents the percentage of CD117^+^CD41^-^, CD117^+^CD41^+^, and CD117^-^CD41^+^ cells in each group; **(C-E)** Flow cytometry analysis of CD41 and CD61 expression in the BM and spleen of each group after treatment for 10 days. The histogram represents the percentage of CD41^+^CD61^+^ cells in each group; **(F-G)** Representative images of VWF immunohistochemical staining of BM after treatment for 10 days. Bars: 100 µm (top), 50 µm (bottom); The histogram shows the number of BM MKs in each group; **(H-I)** Representative images of VWF immunohistochemical staining of spleen after treatment for 10 days. Bars: 100 µm (top), 50 µm (bottom); The histogram shows the number of SP MKs in each group;** (J-L)** PI staining in BM or spleen CD41^+^ cells was used to analyze the DNA content (2, 4 and ≥ 8N) by flow cytometry after treatment. The histogram represents the percentage of 2 N, 4 N and ≥ 8 N cells of BM CD41^+^ cells of each group; **(M-O)** PI staining in lung CD41^+^CD42d^+^ cells was used to distinguish between megakaryocytes and platelets by flow cytometry after treatment. The histogram represents the percentage of CD41^+^CD42d^+^ cells among lung cells and the percentage of megakaryocytes and platelets among lung CD41^+^CD42d^+^ cells in each group. All data represents the mean ± SD of three independent experiments; ^*^p < 0.05, ^**^p < 0.01, ^***^p < 0.001, vs the model group; ^#^*p* < 0.05, ^##^*p* < 0.01, ^###^*p* < 0.001, vs the normal group. Gen: genistin.

Since the hematopoietic system has been reported to be highly sensitive to IR, in particular, IR doses above 2 Gy can lead to myelosuppression, which is characterized by neutropenia, lymphopenia, and thrombocytopenia 45. And combining data *in vivo* and *in vitro*, we sought to determine whether the platelet recovery caused by increased platelet production. Firsty, to dissect the role of genistin in the Mk-P generation process, we sorted CD117(c‑Kit)^+^CD41^-^ (hematopoietic progenitor cells), CD117^+^CD41^+^ (megakaryocytic progenitor cells), and CD117^-^CD41^+^ (MKs) in mouse bone marrow on day 10 by FACS (Figure 5A-B). The results showed that CD117^+^CD41^-^ and CD117^+^CD41^+^ cells were elevated relative to the model group, suggesting that the increase in the number of platelets induced by genistin may be caused by promoting hematopoietic progenitor cell recovery and the production of more megakaryo progenitor cells. In addition, the expression of the bone marrow megakaryocyte markers CD41 and CD61 in the genistin and TPO-treated groups was significantly higher than that in the model group, further indicating that genistin promoted MK differentiation of BM (Figure 5C and D).

The spleen is now viewed as the prominent site of extramedullary hematopoiesis (EMH) 46. Therefore, we also measured the expression of CD41 and CD61 in the spleen, and the results showed that the expression of the genistin and TPO treatment groups was significantly higher than that of the model group (Figure 5C and E). Since both megakaryocytes and platelets in the spleen express CD61 and CD41, we performed cell morphology and immunohistochemical staining (VWF) of the bone marrow and spleen to further clarify whether bone marrow hematopoiesis and EMH coexist. The results showed a significant increase in the number of BM MK in the genistin and TPO-treated mouse groups compared to the model mice (Figure 5F-G). Similar results were observed in the spleen (Figure 5H-I). Meanwhile, the results of H&E staining were also consistent with the above (Figure S2A-D). The above data suggest that genistin can promote megakaryocyte production in the bone marrow and spleen. Since platelets are produced by mature megakaryocytes, we tested megakaryocytes in bone marrow and spleen and found that genistin promoted the formation of polyploidy megakaryocytes (Figure 5J-L). Studies have reported that the lung can capture megakaryocytes and is the main site of terminal platelet production. In addition, the lung is considered an ideal bioreactor for the production of mature platelets 47, 48. Therefore, we explored whether genistin can stimulate lung hematopoiesis. CD42d is a surface marker specific to platelets and megakaryocytes. At the same time, platelets are fragments of nonnuclear megakaryocytes, so we performed three stains of CD41, CD42d and PI to detect the content and proportion of platelets and megakaryocytes in the lungs. Flow cytometry found a higher percentage of CD41^+^CD42d^+^ cells (megakaryocytes and platelets) in the genistin group compared to the model group (Figure 5M-N). This suggested an increased total number of both megakaryocytes and platelets in the genistin group. However, subsequent PI reanalysis within the CD41^+^CD42d^+^ population revealed similar proportions of megakaryocytes and platelets between the genistin and model groups (Figure 5M). Interestingly, since the overall cell counts (CD41^+^CD42d^+^ cells) were higher in the genistin group whereas similar proportions of megakaryocytes and platelets in the total cell counts (CD41^+^CD42d^+^ cells) (Figure 5M), we can infer a relatively increased presence of both megakaryocytes and platelets compared to the model group (Figure 5O). This suggests that genistin may promote lung capture of megakaryocytes, potentially leading to enhanced platelet production within the lung.

Taken together, these data suggest that genistin can promote megakaryocytes in the bone marrow and spleen to produce and mature, and it may also promote the capture of megakaryocytes by the lungs, which can rupture to produce platelets.

### Genistin enhances the function of platelets

**Figure 6:**
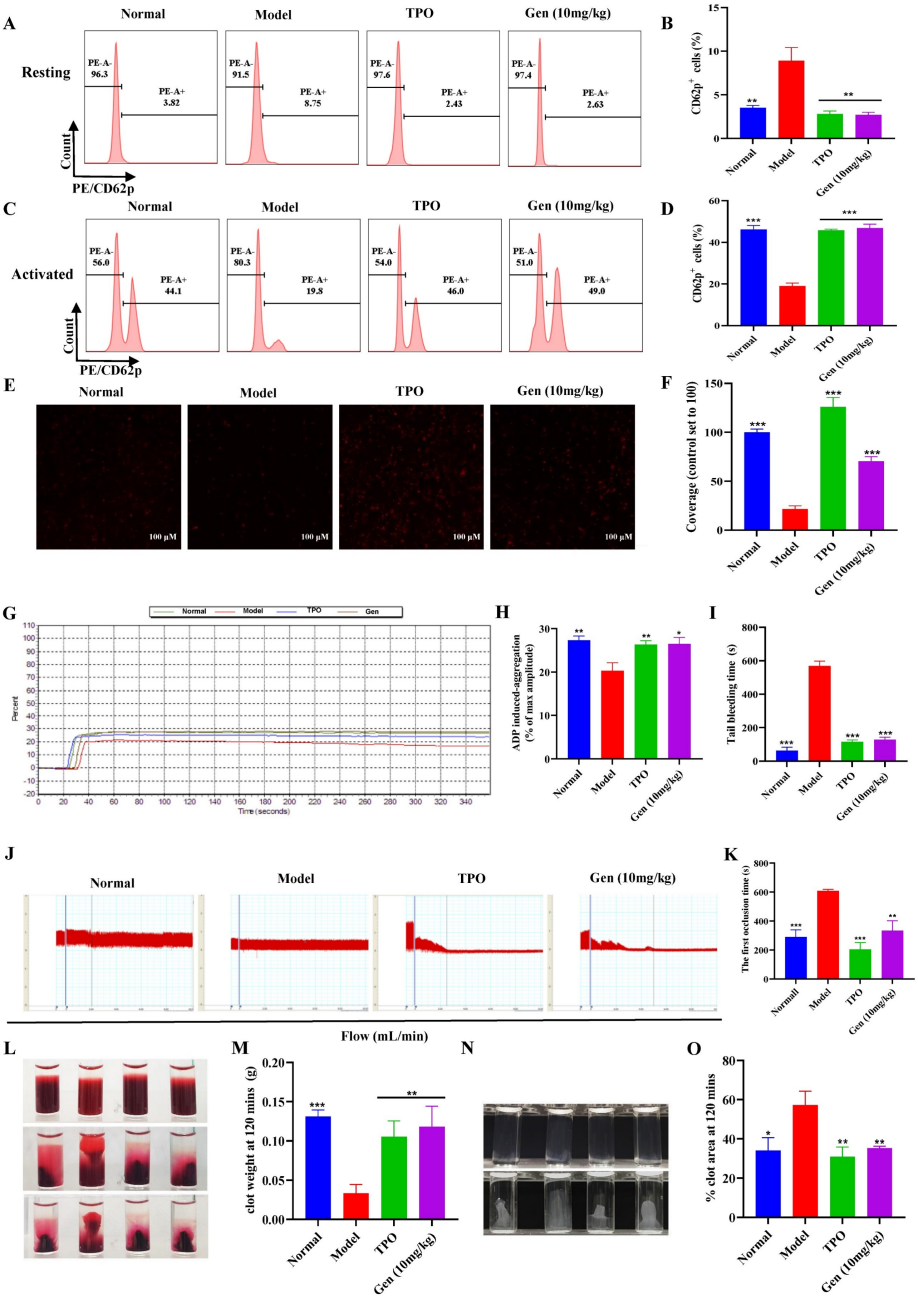
** Genistin enhances the function of platelets; (A)** Representative images of CD62p in washed platelets in each group; **(B)** Statistical analysis results of CD62p ratio in washed platelets without ADP loading in each group; **(C)** Representative images of CD62p with ADP (10 µM) washed platelets in each group; **(D)** Statistical analysis results of CD62p ratio in washed platelets with ADP (10 µM) loading in each group; **(E-F)** Micrographs of collagen-coated slides with the same number of platelets perfused. The red represents platelets. The histogram shows the average coverage of red fluorescence on the whole surface by ImageJ software. **(G-H)** Under the stimulation of ADP, platelet aggregation was measured by a turbidimetric aggregation-monitoring device. The histogram shows the maximum aggregation amplitude of platelets in each group. **(I)** The statistical chart represents the tail clotting time of each group. **(J-K)** Carotid blood flow tracings of each group. Analysis of the first occlusion time of FeCl_3_-induced carotid artery thrombosis formation; **(L-M)** Whole blood from each group of mice, and clot retraction is displayed at the indicated time point; **(N-O)** PLTs from each group of mice, and clot retraction is displayed at the indicated time point; All data represents the mean ± SD of three independent experiments; ^*^p < 0.05, ^**^p < 0.01, ^***^p < 0.001, vs the model group. Gen: genistin, Ctrl: control.

To further verify that genistin-induced platelets are effective, we measured platelet function. Platelets in circulating blood are generally in a "quiescent" state. Therefore, to fulfill particular functions, platelets must first be activated. CD62p (platelet surface P-selectin) is a platelet α granular membrane glycoprotein that is a sensitive marker of platelet activation 49. Activation of platelets is required for a variety of aspects of hemostasis including platelet aggregation, clot retraction, and stable thrombus formation 50. Therefore, we first evaluated the expression of genistin-induced platelet formation in ADP-induced CD62p using flow cytometry detection of PE-conjugated CD62p binding to platelets. The results demonstrated that the expression of CD62p on the surface of the model group was higher at rest relative to other groups (Figure 6A-B). After ADP stimulation, CD62p expression on the platelet surface of mice in the control group and drug intervention group was significantly increased, while CD62p expression in the model group was weaker than that in the other groups (Figure 6C-D). In addition, we measured platelet activation using genistin as the platelet agonist. We incubated washed platelets from normal mice with genistin or vehicle control for 30 minutes followed by platelet activation assays. The result indicated no activation was apparent after stimulation with genistin (Figure S2E-F). This strengthens our hypothesis that genistin primarily exerts its positive effect on hemostasis in pathological conditions such as thrombocytopenia. CD62p expression levels were strongly associated with platelet adhesion, and as expected, platelet adhesion was improved in the genistin and TPO groups (Figure 6E-F) 51. Meanwhile, the aggregation effect of ADP and platelet adhesives on solidified collagen has been studied. The results showed that ADP-induced platelet aggregation was significantly enhanced after genistin treatment compared to the model group (Figure 6G-H). Tail bleeding and FeCl_3_-induced carotid thrombosis models have also been used to verify whether genistin affects hemostasis and thrombosis. Unsurprisingly, both tail-bleeding time (Figure 6I) and first occlusion time of FeCl3-induced carotid artery thrombosis formation (Figure 6J-K) were shorter in the TPO group and genistin group than in the model group. In addition, platelet-driven clot contraction is important for promoting clot stability and for maintaining blood vessel patency 52, 53. Microscopic studies have shown that the main mechanism is that during clot formation, platelets emit filamentous pseudopodia, and longitudinal fine actin filaments contact and "pull" fibrin fibers and "pull" on fibrin fibers, resulting in serum excretion, decrease of clot size, an increase in clot density 54, 55. Therefore, we performed a clot retraction extension experiment. Whole blood clot contraction results showed that clot contraction was also enhanced in the genistin and TPO groups (Figure 6L-M). Similar results were observed in the platelet retraction test after washing (Figure 6M-O).

Finally, since we have demonstrated that genistin has a positive effect on platelet production and function but to ensure its safety. We selected the genistin group with the best efficacy and performed H&E staining and organ indices for major organs, including the liver, lung, kidney, and thymus to assess the safety of genistin use. The results showed that genistin had no significant effect on the main organs at this concentration (Figure S2). All of these findings suggest that genistin has some therapeutic potential for thrombocytopenia caused by radiation damage.

### Genistin interacts with ERβ to regulate megakaryocyte differentiation

Since genistin is known as an estrogen analogue, we next explored whether genistin promotes megakaryocyte differentiation and platelet production via regulation of the ER. ER is mainly divided into two categories, namely ERα and ERβ, so we first used molecular docking to compare the affinity between genistin and the two types of receptors to further judge the potential of genistin and its core target. Docking scores > 5 kcal mol^-1^ were considered high binding intensity.

**Figure 7:**
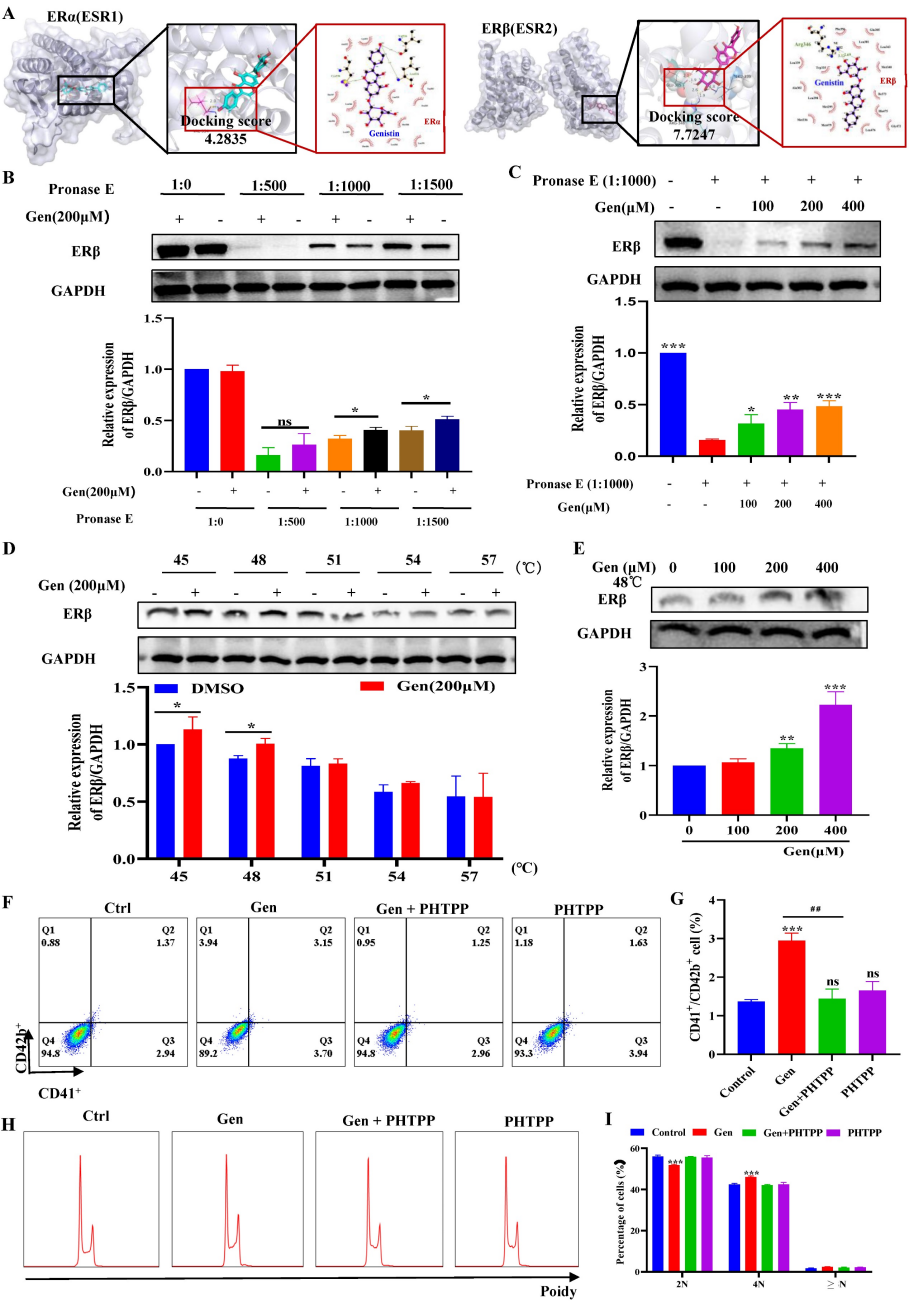
** Genistin interacts with ERβ to regulate megakaryocyte differentiation. (A)** ERα or ERβ and ligands (genistin) by molecular docking. **(B)** Lysates from Meg-01 cells were incubated with or without genistin (200 μM) for 2 h, and different concentrations of pronase E (1:500, 1:1000, or 1500) were added for 10 min at 40°C, ERβ content was detected using western blot analysis. **(C)** Lysates from Meg-01 cells were incubated with genistin (100, 200, 400 μM) for 2 h, and a final concentration of pronase E (1:1000) was added for 10 min at 40 °C. ERβ expression was detected by western blot analysis. **(D)** Lysates from Meg-01 cells were incubated with genistin (200 μM) for 9 h, then reacted at different temperatures (45, 48, 51, 54, 57 °C) for 5 min, and finally the ERβ content was detected by western blot. **(E)** Lysates from Meg-01 cells containing or without different doses of genistin (100, 200, 400 μM) were incubated for 9 h, and then reacted at 48 °C for 5 minutes, and finally the ERβ content was detected by western blot. **(F-G)** Meg-01 cells were detected by flow cytometry using genistin (20 μM), PHTPP (20 μM), and both, respectively. The histogram shows the percentage of CD41^+^CD42b^+^ cells for each group. **(H-I)** PI staining in Meg‑01 cells was used to analyze the DNA content (2, 4 and ≥ 8 N) by flow cytometry after genistin (20 μM), PHTPP (20 μM), and both interventions, respectively. All data represents the mean±SD of three independent experiments; ^*^p < 0.05, ^**^p < 0.01, ^***^p < 0.001, vs the Ctrl group;^ #^*p* < 0.05, ^##^*p* < 0.01, ^###^*p* < 0.001, vs the Gen group; Gen: genistin, Ctrl: control.

Based on the docking part, the binding fraction of ERβ and genistin is 7.7247, but the binding fraction with ERα is 4.2835, which indicates that genistin has a good affinity for ERβ, so we speculate that there is a direct interaction between genistin and ERβ (Figure 7A). Then, we employed a DARTS approach to further confirm this direct interaction of genistin with ERβ, because this assay identifies direct targets for the ligand of interest without additional modification 56, 57. DARTS detects ligand-bound targets in lysates based on their increased resistance to proteolysis. The test can detect changes in stability only if a high concentration of ligand is required for the assay in the cell lysate after proteolytic digestion 57. After exposure of Meg-01 cell lysate to 200 μM genistin for 4 h, ERβ levels were higher than those in the untreated group under different protease E conditions (Figure 7B). This effect has also been shown to be dose dependent, likely due to the resistance to pronase degradation (Figure 7C). In addition, we would like to go further and prove it through an orthogonal target verification method, CETSA. CETSA identifies ligand-target ligands by immunoblotting detection of increased stability of the target proteins toward heat-induced precipitation. As expected, genistin incubation stabilized ERβ in thermally denatured Meg-01 cell lysates (Figure 7D-E). The above data suggest that genistin may act as a ligand for ERβ. Finally, to clarify that genistin promotes promegakaryocyte differentiation produced by acting on ERβ, we administered an ERβ inhibitor (PHTPP). The results showed that PHTPP could indeed block the expression of CD41^+^CD42b^+^ produced by genistin, and its expression alone was not different from that of the control group (Figure 7F-G). Similar results were observed in the cell ploidy assay (Figure 7H-I). Collectively, these results suggest that genistin directly interacts with ER in Meg-01 cells and indicate that ERβ is a potentially important target of genistin to promote MK differentiation and platelet production.

### Genistin activates ERβ/PI3K/AKT and ERβ/MEK/ERK to promote megakaryocyte differentiation

**Figure 8:**
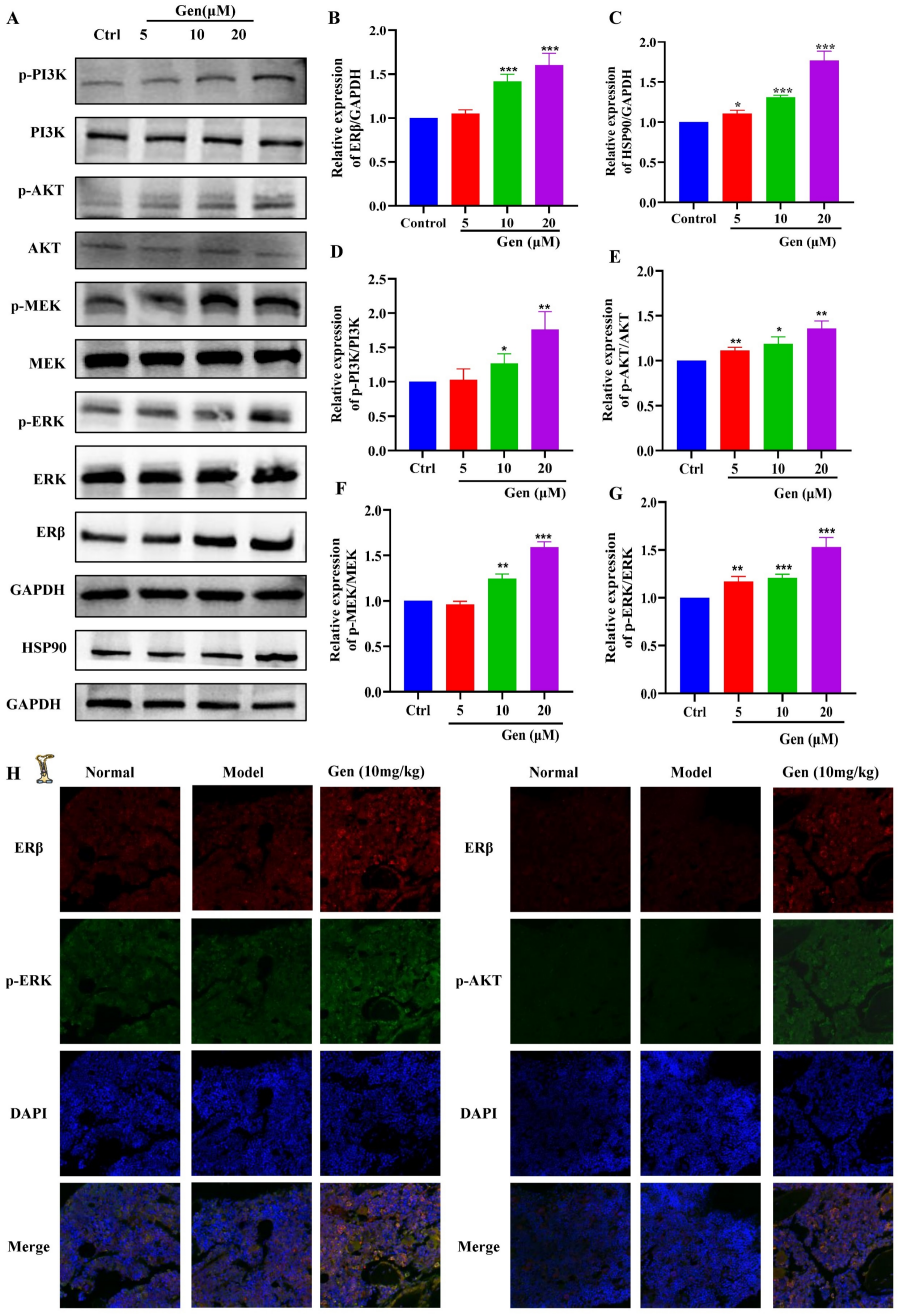
** Genistin can promote PI3K/AKT and MEK/ERK expression. (A-G)** The expression levels of ERβ/GAPDH, HSP90/GAPDH, p-PI3K/PI3K, p-AKT/AKT, p-MEK/MEK, and p-ERK/ERK were detected by western blot after Meg-01 cells were treated with genistin for 5 days. **(H)** Representative immunofluorescence images of ERβ fluorescently double-stained with p-ERK and p-AKT, respectively, on bone marrow cells after 10 days of treatment with genistin in a mouse model of radiation thrombocytopenia. All data represents the mean ± SD of three independent experiments; ^*^p < 0.05, ^**^p < 0.01, ^***^p < 0.001, vs the Ctrl group; Gen: genistin, Ctrl: control.

**Figure 9:**
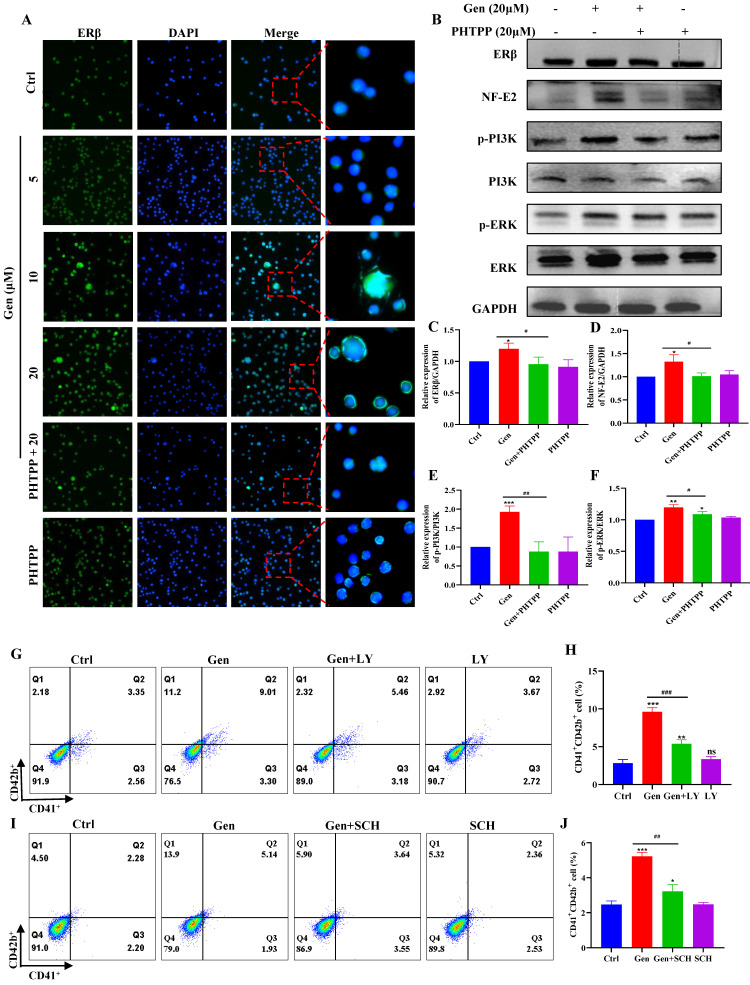
** Genistin promotes megakaryocyte differentiation through the activation of PI3K/AKT and MEK/ERK. (A)** Representative immunofluorescence images of the nuclear translocation of ERβ in Meg‑01 cells upon treatment with genistin for 5 days. All data represents the mean ± SD of three independent experiments. **(B-F)** The expression levels of ERβ/GAPDH, p-PI3K/PI3K, p-ERK/ERK, and NF-E2/GAPDH were detected by western blot after Meg-01 cells were treated with genistin (20 μM), PHTPP (20 μM) or both intervention for 5 days. **(G-H)** Flow cytometry analysis of CD41^+^CD42b^+^ expression in Meg-01 cells treated with genistin and LY treatments for 5 days. **(I-J)** Flow cytometry analysis of CD41^+^CD42b^+^ expression of Meg-01 cells with genistin and SCH treatments for 5 days. All data represents the mean ± SD of three independent experiments; ^*^p < 0.05, ^**^p < 0.01, ^***^p < 0.001, versus the model group;*^ #^p* < 0.05, ^##^*p* < 0.01, ^###^*p* < 0.001, vs the Ctrl group; Gen: genistin, Ctrl: control; LY: LY294004; SCH: SCH772984.

To further evaluate the potential of genistin in hematopoiesis, a detailed mechanistic understanding of the influence of genistin is required. We wondered whether it could activate ERβ and investigated its effect on the stability of the ERβ/HSP90 complex. In general, ERβ is not activated because most of the ERβ/HSP90 complex exists, and once activated, HSP90 is separated from the cup, so we detected ERβ and HSP90 protein expression. The results showed that the protein expression of both proteins showed a dose-dependent upward trend (Figure 8A-C). After the activation of the ER located in the plasma membrane, it has been reported it can activate the kinase cascade of PI3K 59, mitogen-activated protein kinase (MAPK) 60, and endothelial nitric oxide synthase (eNOS) activation 61 to initiate rapid signal transduction. Meanwhile, previous studies have suggested that the activation of the ERK1/2 and Akt pathways is strongly associated with thrombopoiesis. With the use of the previously mentioned data, we measured the expression of PI3K/AKT and MEK/ERK signaling pathway proteins. The results showed that, as expected, both pathway protein expressions showed concentration-dependent upregulation (Figure 8A and 8D-G). To further clarify that gensitin can activate PI3K/AKT and MEK/ERK signaling pathways through ERβ. We performed two sets of immunofluorescence staining for ERβ/p-PI3K and ERβ/p-ERK on bone marrow sections from gen-treated thrombocytopenia mouse. The results showed that ERβ and p-PI3K and p-ERK in the genistin group could be expressed on the same cells, while the fluorescence expression of those was higher than that in the model group (Figure 8H). This was consistent with the *in vitro* assay and further confirmed our hypothesis. However, estrogen receptors can be distributed in the cell membrane, cytoplasm, so in order to distinguish between genistin megakaryocytic cell differentiation is induced by membrane receptor activation or nuclear activation. Therefore, we used immunofluorescence methods to detect changes in the expression of ER in cells. The results showed that with the increase of genistin concentration, the fluorescence intensity of ERβ in the nucleus and the cytoplasmic membrane increased (Figure 9A). Therefore, we hypothesized that genistin could act through two distinct mechanisms: activating membrane ERβ to trigger PI3K/AKT and MEK/ERK signalings, and directly activating nuclear ERβ to promote transcription. This dual action, we proposed, would synergistically contribute to enhanced platelet production.

To further verify that genistin activates PI3K/AKT and MEK/ERK to produce megakaryocytes differentiation through ERβ, we administered ERβ inhibitors to redetect their protein changes. The results demonstrated that protein expression of PI3K, ERK and NF-E2 was indeed blocked by ERβ inhibitors (Figure 9B-F). After that, we continued to administer PI3K inhibitors to detect the prodifferentiation results of gensitin, and found that after coincubation of genistin and PI3K inhibitors, CD41^+^CD42b^+^ expression decreased compared to the genistin group, but its expression was still higher than that of the control group (Figure 9G-H). The same results were found with ERK inhibitors (Figure 9I-J). The above results further indicate that genistin activates the membrane ERβ and activates the PI3K/AKT and MEK/ERK signaling pathways.

## Discussion

Platelets are pivotal in primary hemostasis and possess nonhemostatic properties involved in angiogenesis, tissue repair, inflammation and metastatis 62. The platelet count is a contributor to these pathophysiological processes. Because once the number of platelets decreases, patients with hematologic disorders and thrombocytopenia have an increased risk of bleeding. Therefore, in order to avoid bleeding, a lot of effort has been invested in prevention strategies, mainly focusing on improving platelet count. The processes of megakaryopoiesis and thrombopoiesis can affect platelet production. Megakaryogenesis is a continuous process of hematopoietic stem cell commitment to the MK lineage that involves the proliferation of progenitor cells and maturation of MKs, and ends with the release of PPFs and platelets. In addition, thrombocytopenia in bone marrow injured states is a significant concern post-chemotherapy or radiation exposure. Meanwhile, in radiation injury, platelet count drop correlates with risk of death. However, each of the thrombocytopenia therapies available today has its disadvantages, such as poor treatment of TPO agonists for TPOR-deficient humans and traumatic surgical treatment 5, 63. Therefore, in this setting, development of an agent that is able to significantly improve nadir platelet counts and speed count recovery is of vital importance.

Machine learning (ML) is a branch of artificial intelligence (AI) that is most commonly used in the pharmaceutical industry for the virtual screening of active compounds, which greatly reduces the time spent on traditional drug screening 64, 65. In this study, we used model-building methods that have been reported in research, randomForest, which was used to establish a screening model to predict the drug screening model of natural compounds with hematopoietic activity and explore the potential therapeutic active compounds in the compound library for the treatment of thrombocytopenia. In this process, we discovered a highly reactive compound, genistin, which may play a role in the treatment of thrombocytopenia (Figure 1). Genistin is found in a variety of dietary plants. Current pharmacological activity studies of genistin show that it has anticancer and anti-inflammatory activities. Pharmacological activity related to hematopoietic processes has not yet been studied. Since megakaryocyte maturation is crucial for platelets, we first tested the effect of genistin in promoting megakaryocytes differentiation *in vitro*. It was found that genistin did have a potential effect on megakaryocyte differentiation within a certain concentration range (Figure 2). Meanwhile, we also tested the toxicity of its differentiation concentration *in vitro* and found that it was not toxic, and the above results showed that genistin can be used as an inducer of megakaryocytes differentiation and has potential for the treatment of thrombocytopenia (Figure S1).

However, the degree of differentiation has certain limitations, as we used the human megakaryocyte model and the source of platelet precursors (Meg-01 and K562) rather than primary cells to study their differentiation 66, 67. Therefore, we next further verified the effect in a mouse thrombocytopenia model. Here, we found that genistin increases platelet levels in a mouse model of thrombocytopenia without a significant effect on other cells (Figure 4A-G). Also, it is worth noting that platelet counts did not rise in the genistin group when genistin was continued treatment after platelet counts returned to normal (Figure 4B). The results were consistent with the platelet count results of genistin-given to normal mice (Figure 4H). This strengthens our hypothesis that genistin exerts its positive effects mainly on the pathological conditions of thrombocytopenia and has no effect on platelets in normal mice, thus reducing the risk of thrombosis. These data further show that genistin has better and safer therapeutic potential for thrombocytopenia models (Figure 4B and H-I). The fine regulation of platelet production and sequestration/destruction is critical to maintain steady platelet counts. Considering that the platelet model we used was radiation-based, the bone marrow is most easily damaged by radiation 45. So we first examined the changes in progenitor cells in the bone marrow. The result shows that c-Kit^+^CD41^-^ (hematopoietic progenitor cells) and c-Kit^+^CD41^+^ (megakaryocytes progenitor cells) cells were elevated relative to the model group, suggesting that the increase in the number of platelets induced by genistin may be caused by promoting hematopoietic progenitor cell recovery and the production of more megakaryo-progenitor cells (Figure 5A-B). Later, considering that bone marrow and spleen are important hematopoietic locations, we performed Immunohistochemical staining of VWF, H&E staining and flow cytometry for megakaryocyte markers, and the results showed that the content or expression level was increased relative to the model group, and the megakaryocyte maturation test was similar 68 (Figure 5C-L). Recent studies have shown that the lung is also the main site of end-stage platelet production and a reservoir of hematopoietic progenitor cells 47. Considering that the lungs are important organs of systemic circulation and rich in platelets, most megakaryocyte markers can also be expressed on platelets. Therefore, we performed triple staining using whether the two contained nuclei to study the effect of genistin on lung megakaryocytes and platelets. It was found that relative percentage of the lung CD41^+^CD42d^+^ cells (megakaryocytes and platelets) were higher in the genistin group and TPO group compared to the model group, and PI reanalysis of CD41^+^CD42d^+^ cells showed that the proportion of platelets and the proportion of megakaryocytes in the genistin group was similar to that in the model group. Therefore, it can be inferred that the relative percentages of megakaryocytes and the relative percentages of platelet in the gensitin group are higher than those of the model, indicating that genistin can promote lung capture of megakaryocytes, thereby increasing the potential of platelet number (Figure 5M-O). Then, thrombocytopenic mice experience platelet dysfunction due to low platelet count and platelet injury induced by irradiation, potentially increasing their bleeding risk. Therefore, in order to verify the functional nature of platelets produced by genistin-induced differentiation, we conducted a series of platelet function testing experiments, such as platelet activation/aggregation/adhesion, plaque retraction, and carotid artery thrombosis. All of the above experiments show that genistin's ability to restore platelet function in this model suggests a beneficial effect on hemostasis under pathological conditions (Figure 6A-O). In contrast, the absence of activation observed washed platelets from normal mice indicates that genistin is unlikely to act as platelet agonist to activate platelett in healthy individuals (Figure S2E-F), thus mitigating concerns about potential thrombotic risks. All *in vivo* results show that the effect of genistin on hematopoiesis is multifaceted and multisite, suggesting that the potential for clinical use of genistin in the treatment of RIT.

Transcription factors (TFs) are proteins that bind to specific DNA sequences and regulate expression of genes 69. Its expression affects diverse aspects of megakaryocyte biology, and platelet production and function, culminating in thrombocytopenia and platelet dysfunction. For example, GATA1 knock-out mice display thrombocytopenia accompanied by an increase in the number of MKs that are characterized by decreased polyploidization and a defect in cytoplasmic maturation 70. Mice lacking p45 NF-E2 show profound thrombocytopenia resulting from a late arrest in megakaryocyte differentiation, and a number of red blood cell defects, including anisocytosis and hypochromia 71. Here, TF presentation for gensitin interventions increased with increasing concentration, which further demonstrated the role of genistin in megakaryocyte differentiation (Figure 3A-F). Therefore, to further evaluate the potential of genistin in hematopoiesis, a detailed mechanistic understanding of the influence of genistin is required. It has previously been reported that MK polyploid formation can be regulated by ERβ signaling 71. In addition, small amounts of estrogen can promote upregulation of platelet levels, and genistin is an estrogen analogue 10, 72, 73. Therefore, we further clarify whether platelet formation is related to estrogen receptors. We first screened ERα (ESR1) and ERβ (ESR2) by molecular docking. The results showed that ERβ had a high affinity. Next, according to the order of affinity, we used DARTS and CETSA experiments for detection, and the results showed that further verified our conjecture that ERβ may be a target for genistin. Finally, loading ERβ-target inhibitors was found to block the MK differentiation promotion effect of genistin. This further proves that ERβ is the main target of genistin to promote MK differentiation (Figure 7A-I). Notably, the growing amount of evidence for modulation of intracellular pathways by estrogen receptors, such as the MAPK pathway, the PI3K. It has also been reported that membrane ERβ triggers secondary signaling such as ERK or PI3K activation 75, 74, 76. Therefore, we speculate that the binding of genistin to ERβ may trigger downstream signaling pathways that promote MK differentiation and platelet formation. We examined the effect of genstin on the protein expression of PI3K/AKT and MEK/ERK signaling pathways, and found that it showed an up-regulated trend (Figure 8A-G). Meanwhile, ERβ blockade resulted in a corresponding decrease in the levels of p-ERK and p-AKT (Figure 9B-F). In addition, genistin-induced megakaryocyte differentiation can be attenuated by PI3K inhibitors and ERK inhibitors (Figure 9G-J). This is sufficient to demonstrate that genistin promotes megakaryocyte differentiation through ER activation of PI3K/AKT and MEK/ERK pathways. However, ERs are members of a large superfamily of NRs. These receptors act as ligand-activated transcription factors. The classical mechanism of ER action involves estrogen binding to receptors in the nucleus, after which the receptors dimerize and bind to specific response elements known as estrogen response elements (EREs) located in the promoters of target genes, causing transcription to occur 58. Studies have reported that previous studies have been conducted on the classical genetic pathways that promote platelet production. Of note, ERβ in this study is also a nuclear transcription factor. However, it is well known that ERβ can exist in the cytoplasm, nucleus and cell membrane. Estrogen-mediated signaling events can be divided into genomic and non-genomic events. Non-genomic effects involve indirect regulation of gene expression through various intracellular signaling events. Therefore, we performed immunofluorescence staining and found that the fluorescence of ERβ has an enhanced effect on the expression of both the nucleus and the membrane, so we speculate that genistin activates both the nuclear ERβ signaling pathway and the membrane ERβ signaling pathway (Figure 8H and Figure 9A). Therefore, in this study, we hypothesized that genistin could activate the membrane signaling receptor ERβ, which in turn activates PI3K/AKT and MEK/ERK, and that genistin could also synchronously and directly activate ERβ, which in turn enters the nucleus and causes transcription. Together, they promote platelet production.

**Figure 10:**
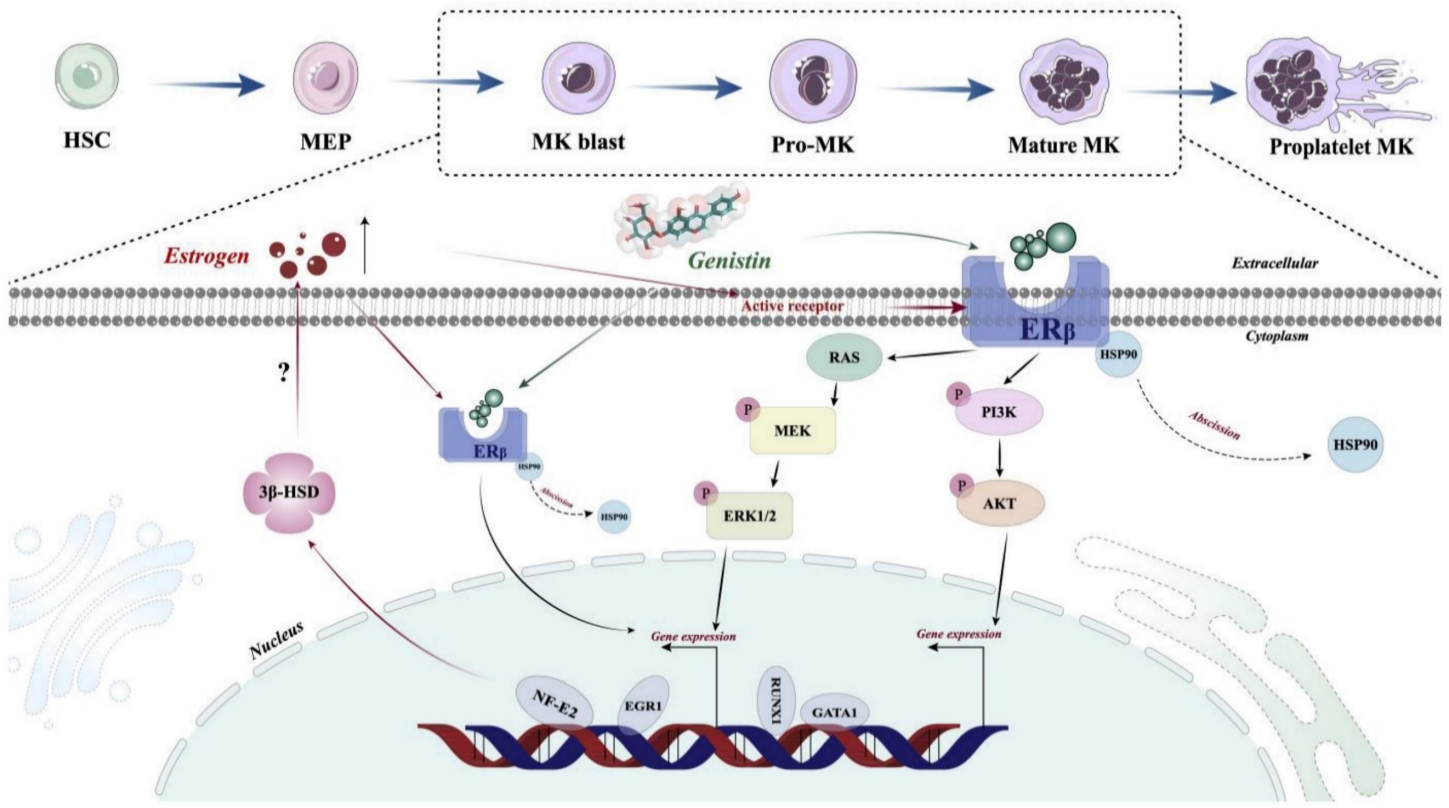
** Schematic illustration of the role of genistin in regulating thrombopoiesis.** Genistin stimulates the activation and translocation of ERβ to the nucleus of MK and then directly initiates transcription factor transcription. The expression of GATA1 and NF-E2 is upregulated, which will promote polyploidy and proplatelet formation, respectively. At the same time, upregulation of NF-E2 can induce the production of 3β-HSD, an enzyme involved in estrogen synthesis, which in turn may further promote polyploidy MKs and proplatelet formation. In addition, genistin can directly stimulate membrane ERβ, which in turn activates PI3K/AKT and MEK/ERK signaling pathways, which in turn causes transcriptional effects.

Although in this study, we demonstrated that the estrogen analog genistin promotes platelet production by activating the ERβ/PI3K/AKT and ERβ/MEK/ERK pathways. However, there are still shortcomings in this study that need to be further verified. First, we failed not use human primary cells (CD34^+^ cells and iPSCs) to study the effect of genistin on megakaryocyte differentiation. Secondly, ERβ is primarily a nuclear receptor, and in the results of immunofluorescence, we found that the fluorescence expression of ERβ in the nucleus also increased with increasing genistin concentration. Therefore, whether genistin can directly activate ERβ, and then enter the nucleus to cause transcription effects, which also needs further investigation. Third, in target-related experiments, we found that genistin's direct target is ERβ, but it is interesting that ERβ protein expression showed a trend of upregulation. It was reported that MKs themselves can synthesize estradiol through 3β-hydroxysteroid dehydrogenase (3β-HSD) driven by NF-E2, and estradiol can also work as a mediator of NF-E2 to promote proplatelet formation 77. Therefore, we hypothesize that genistin-induced upregulation of GATA1 and NF-E2 expression during megakaryocyte differentiation may in turn promote estrogen production by increasing the expression of 3β-HSD in MKs (Figure 10).

However, this requires more experimentation for verification. Meanwhile, based on the mechanism of ERβ upegulation induced by genistin described above, this study elucidates the potential of ERβ on platelet production, which may provide a new direction for clinical basic research of thrombocytopenia. Therefore, elucidating these problems is currently a research priority in our future experiments.

This study demonstrates genistin has the capacity to act directly on ERβ and ameliorate the thrombocytopenia response after radiation injury by affecting the platelet production process via ERβ/PI3K/AKT and ERβ/MEK/ERK. This effect of genistin promoted the regeneration of hematopoietic progenitor cells, increased the ability to differentiate and mature to megakaryocytes, and increased the number of platelets. In addition, genistin can improve the hematopoietic function in mices after injury. Based on our data both *in vivo* and *in vitro*, it is reasonable to believe that genistin treatment could be a potential treatment strategy for thrombocytopenia. Meanwhile, the findings of this study provide a theoretical foundation and fresh perspective for the development of new thrombocytopenia treatments.

## Supplementary Material

Supplementary figures.

## Abbreviations

MK: megakaryocyte
PPF: platelet formation
TPO: thrombopoietin
CAMT: congenital amegakaryocytic thrombocytopenia
HSC: hematopoietic stem cell
MEP: megakaryocyte-erythroid progenitor
Mpl: thrombopoietin receptor
AI: artificial intelligence
ERs/ESRs: estrogen receptors
ERα/ESR1: estrogen receptors alpha
ERβ/ESR2: estrogen receptor β
NR: nuclear receptor
MAPK: mitogen-activated protein kinase
DARTS: drug affinity responsive target stability assay
CETSA: cellular thermal shift assay
LDH: lactate dehydrogenase
CCK8: cell counting Kit-8
WBC: white blood cell
RBC: red blood cell
MPV: mean platelet volume
TFs: transcription factors
EREs: estrogen response elements
NFE2: nuclear factor, erythroid 2
GATA1: GATA-binding protein 1
RUNX1: runt-related transcription factor 1
EGR1: early growth response 1
