# Supplementary material for: Genistin: A Novel Estrogen Analogue Targeting ERβ to Alleviate Thrombocytopenia
Source: Int J Biol Sci. 2024 Mar 25;20(6):2236–60. doi: 10.7150/ijbs.90483 (PMC11008259; doi:10.7150/ijbs.90483)
Supplement: Supplementary file 1 — Supplementary figures. [file ijbsv20p2236s1.pdf]

Supplementary Materials for
Safety concentrations of genistin *in vitro* and *in vivo*，and Effect of genistin on H&E staining of bone marrow and normal mouse platelets

Includes: Fig S1; Fig S2; Fig S3;
Supplementary Figure

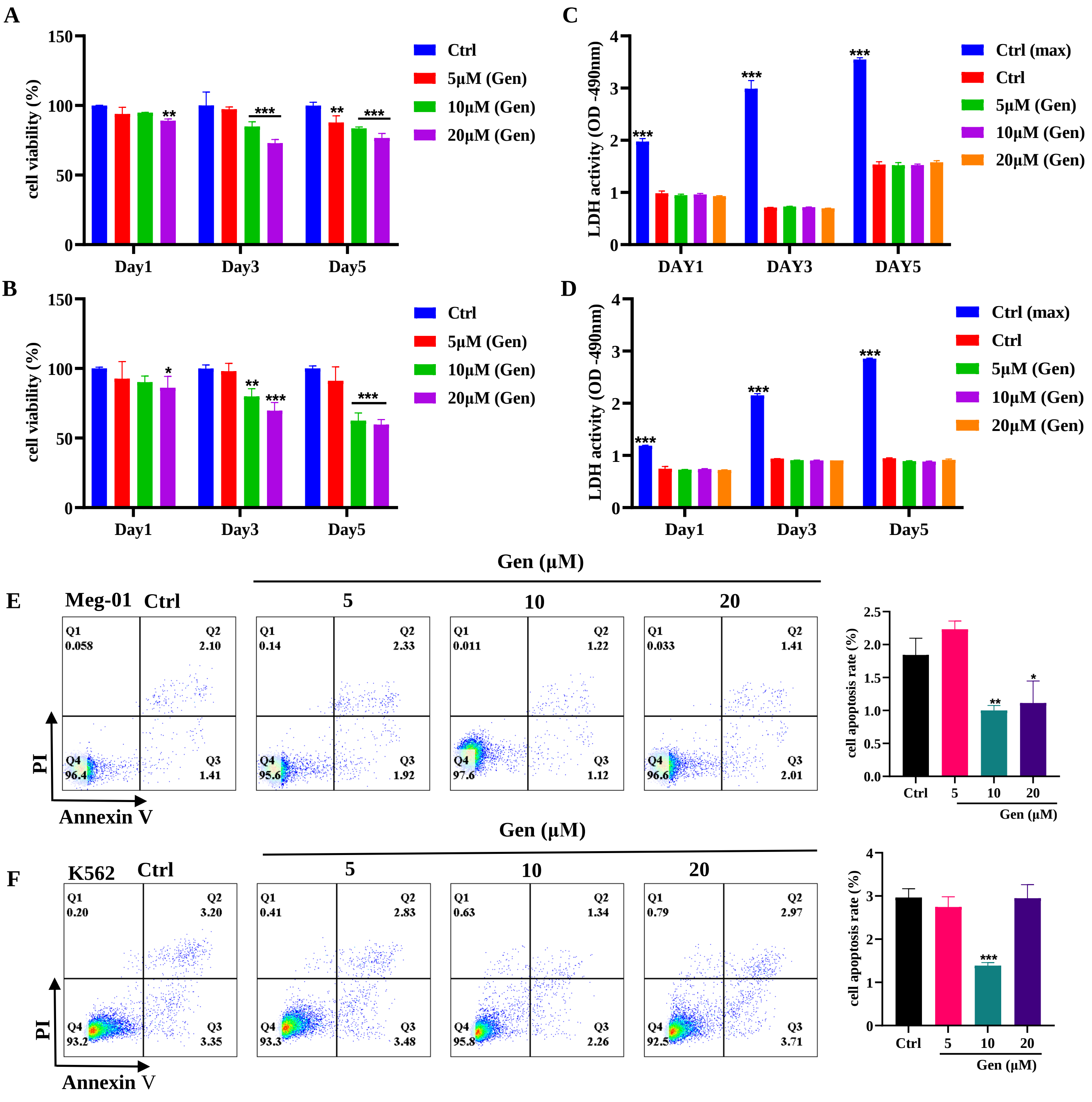

Figure S1. Safety of K562 and Meg01 cell concentrations *in vitro*. (A-B) Measurement of cell proliferation of K562 and Meg-01 cells with the CCK-8 assay after genistin intervention (n=3). (C-D) LDH assay for K562 and Meg-01 cell cytotoxicity after genistin intervention. Ctrl (max) group (sample maximum activity control group) (n=3). (E-F) Apoptosis analysis in K562 and Meg-01 cells after treatment with or without genistin for 5 days. Q2 (Annexin^{+}PI^{+}) denotes late apoptotic (n=3). Q3 (Annexin^{+}PI^{-}) denotes early apoptotic cells. Statistical analysis of apoptotic cells (Q2) of K562 and Meg-01 cells in each group. All data are means ± SD (n=3). $^{*}p < 0.05$, $^{**}p < 0.01$, $^{***}p < 0.001$, vs the Ctrl group. Gen: genistin. LDH: lactate dehydrogenase. Ctrl： control.

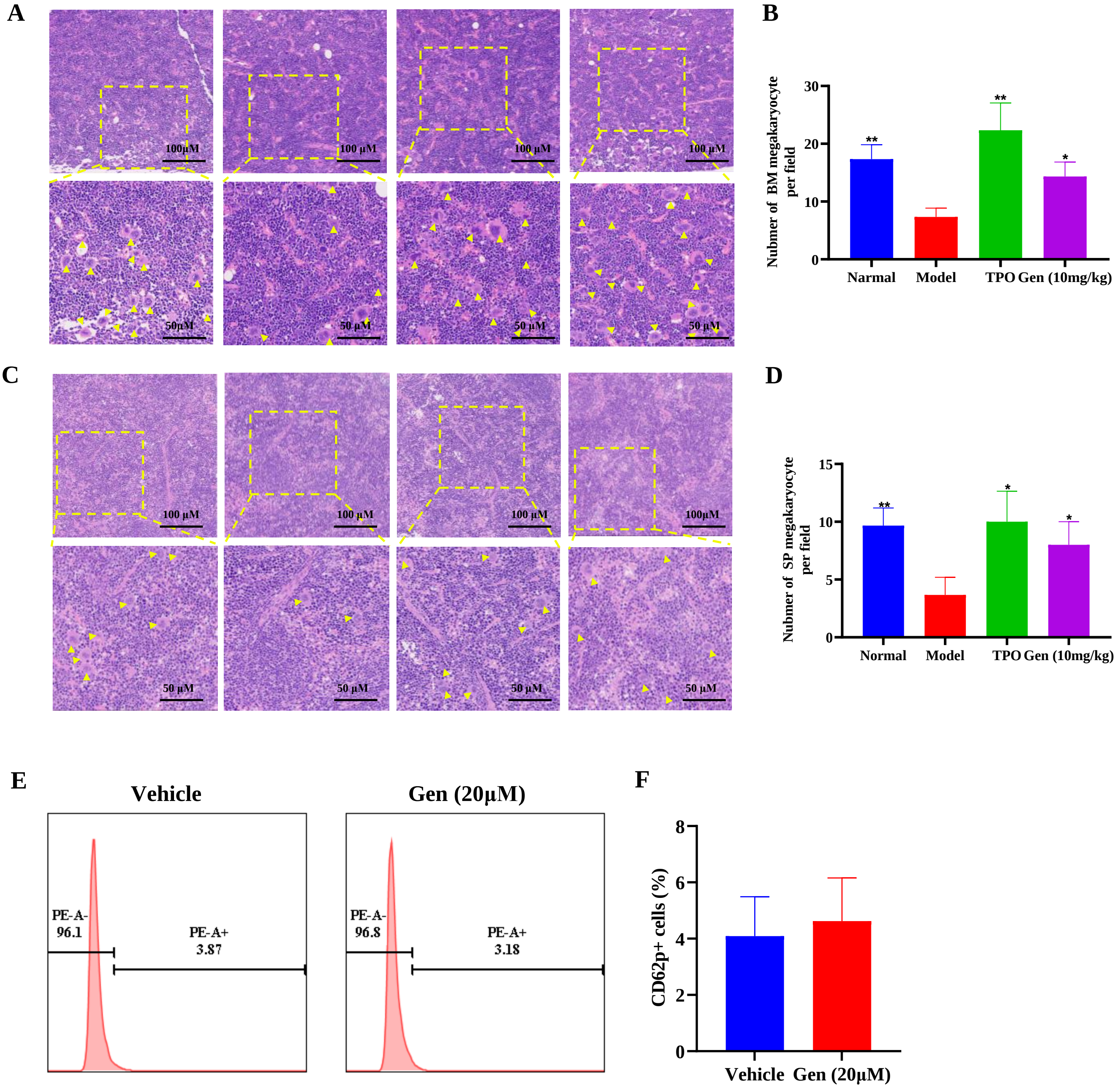

Figure S2. Effect of genistin on H&E staining of bone marrow and normal mouse platelets. (A-B) Representative images of H&E staining of BM after treatment for 10 days. Bars: 100 μm (top), 50 μm (bottom); The histogram shows the number of BM MKs in each group; (C-D) Representative images of H&E staining of spleen after treatment for 10 days. Bars: 100 μm (top), 50 μm (bottom); The histogram shows the number of SP MKs in each group; (E-F) Representative images of CD62p with or without genistin (10 μM) washed platelets in each group; Statistical analysis results of CD62p ratio in washed platelets ith or without genistin (10 μM) loading in each groups; All data represent the mean ± SD of three independent experiments; $^{*}p < 0.05$, $^{**}p < 0.01$, $^{***}p < 0.001$, vs the model group.

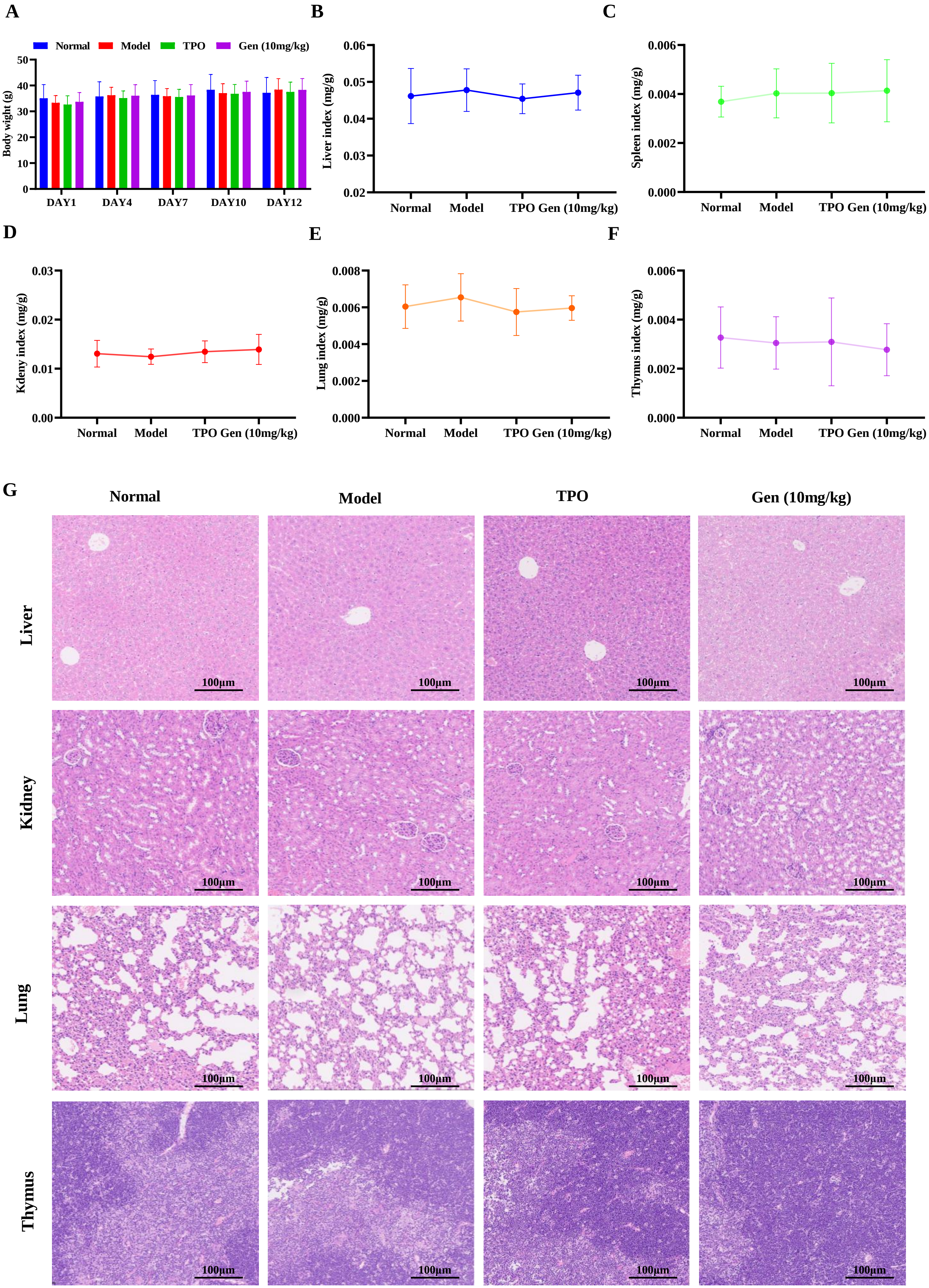

A
Normal
Model
TPO
Gen (10mg/kg)
Body wight (g)
DAY1
DAY4
DAY7
DAY10
DAY12
B
Liver index (mg/g)
Normal
Model
TPO
Gen (10mg/kg)
C
Spleen index (mg/g)
D
Kdeny index (mg/g)
E
Lung index (mg/g)
F
Thymus index (mg/g)
G
Normal
Model
TPO
Gen (10mg/kg)
Liver
Kidney
Lung
Thymus
100μm

Figure S3. Safety of drug concentrations *in vivo*. (A) Body weight in each group on day 0, 4, 7, 10, 12. (B-F) Liver, kidney, spleen, lung and thymus index in each group on day 12. (G) H&E staining of the liver kidney, lung and thymusin each group on day 10. Bars: 100 μm. Data are mean ± SD (n=6, ANOVA). $^{*}p < 0.05$, $^{**}p < 0.01$, $^{***}p < 0.001$, vs the model group. Gen: genistin
